# Effects of Recruiting Midwives into a Family Physician Program on Women's Awareness and Preference for Mode of Delivery and Caesarean Section Rates in Rural Areas of Kurdistan

**DOI:** 10.1371/journal.pone.0151268

**Published:** 2016-04-08

**Authors:** Shayesteh Hajizadeh, Fahimeh Ramezani Tehrani, Masoumeh Simbar, Farshad Farzadfar

**Affiliations:** 1 Midwifery and Reproductive Health Department, Faculty of Nursing and Midwifery, Shahid Beheshti University of Medical Sciences, Tehran, Iran; 2 Reproductive Endocrinology Research Center, Research Institute for Endocrine Sciences, Shahid Beheshti University of Medical Sciences, Tehran, Iran; 3 Non-Communicable Diseases Research Center, Endocrinology and Metabolism Population Sciences Institute, Tehran University of Medical Sciences, Tehran, Iran; Cardiff University, UNITED KINGDOM

## Abstract

**Background:**

The accepted rate rate of caesarean section is 15%. It is expected that an increase in the density of midwives in the family physician program lead to a decrease in this indicator. This study aimed to compare the rates of caesarean section and women's awareness and preference for mode of delivery before and after the implementation of the family physician program in health centres with and without an increase in midwives density.

**Methods:**

In this cross-sectional study, using multistage cluster sampling method a total of 668 mothers with two-month-old children were selected from among all mothers with two-month-old children who were living in rural areas of Kurdistan province. Using the difference-in-differences model and Matchit statistical model, the factors associated with caesarean section rates and women's awareness and preference for mode of delivery were compared in centres with and without an increase in midwives density after the implementation of the family physician program. To compare the changes before and after the program, we used the data collected from the same number of women in 2005 as the baseline.

**Results:**

After adjusting for baseline data collected in 2005, the resutls showed no significant change in caesarean section rates and women's awareness and preference for mode of delivery in the centres with and without an increase in midwives density after the implementation of the family physician program. The Matchit model showed a significant mean increase 14%(0.03–0.25) in women’s awareness of the benefits of natural childbirth between 2005 and 2013 in health centres where the density of midwives increased compared with health centres where it did not. The difference-in-differences model showed that the odds ratio of women’s preference for caesarean section decreased by 41% among participants who were aware of the benefits of natural childbirth, (OR = 0.59, 95% CI: (0.22–0.85); P>0.001).

**Conclusions:**

The results of this study showed that an increase in the density of midwives in the family physician program led to an increase in women's awareness of the benefits of natural childbirth. An increase in women’s awareness of the benefits of natural childbirth was associated with a decreased preference for caesarean section, however this reduction did not have a significant impact on caesarean section rates; possibly, this finding might be attributed to the complexity of this problem that needs a mixed strategy involving various stockholders.

## Background

Caesarean section is a major health issue that can have negative effects on the lives of women of childbearing age[1,2]. Not only are caesarean sections associated with increased risks of morbidity and mortality in mothers and newborns, but also unnecessary caesarean sections impose a high social and economic burden on people and health care systems [3–6, 7].

The World Health Organization reported that a maximum of 15% of deliveries worldwide may be carried out via a caesarean section when proper indications are present [8, 9]. However, caesarean section rates vary among different parts of the world, and this procedure is increasingly performed for various reasons, including increased demand by women for deliveries by caesarean section [10]. The caesarean section rates have also increased significantly in Iran over the past decades, growing from less than 7% in 1970 to 35% in 2000 and reaching 40.7% in 2005, according to a demographic and health survey(DHS) study[11]. At the end of the last decade, the caesarean section rates in Iran increased from 47% to 52% of all deliveries and consists of 64% of all deliveries by private sector providers in Tehran [11, 12]. This rate is much higher than those in many developing and developed countries [13, 14]. These figures from the last three decades represent a six-fold increase in the caesarean section rates for Iran[15].

The conceptual frameworks provided by Freitas and previous studies show that many factors affect the decisions for the choice of delivery in health care centres[16]. The factors related to mothers who receive health services include the following: personal, social, demographic and cultural characteristics; socioeconomic status; type of health insurance; history of delivery; obstetric history; personal choice; and the condition of the mother at the time of admission to the maternity ward and during the birth process. Health service provider factors can include the type of hospital, availability of new medical technologies and clinical staff, staff training and equipment available in the health care centre[16–18].

At the present time, because of the changes to population policies that encourage people to have more children, the caesarean section rates in Iran are associated with great risks and inappropriate consequences[19]. In an analysis of maternal mortality trends from 2001 and 2006 that assessed related risk factors, the results showed that caesarean section usage was one of the factors affecting the mortality of pregnant women in Kurdistan province [20].

Building and strengthening human resource capacity in the health system has always been considered a key solution to health crises in less developed countries[21]. Extensive research has shown that human resources are a clear prerequisite for providing health care; moreover, to conduct many medical interventions there is a need for physicians, nurses, health workers and other health staffs[22].

Several studies have investigated the impact of human resources on primary care and basic prevention services. The continuation of care from a fixed service provider promotes better patient outcomes for primary prevention services[23]. The results of a systematic review conducted in 2006 showed that improvements in health indicators were associated with continuity of care, time of counselling, relationship between family physician and patient and the implementation of preventive activities[24].

Cochrane’s systematic review showed that health care workers could help patients to make better decisions, which could contribute to improved women's awareness about health care options available, increase realistic expectations, reduce conflict and promote an active role of patients in decision-making[25]. Raising the awareness of mothers about natural childbirth and caesarean sections are important components of maternal care; it provides a basis for informed consent and decisions utilizing normal delivery or a caesarean section[26].

In 2005, the family physician program was implemented in rural areas of Kurdistan, resulting in an increase in the density of midwives to improve the prenatal cares through introduction of referral system and providing free of charge insurance system[27].

One of the current topics of interest in the field of reproductive health is determining which factors affect caesarean section decisions, including the use of human resources to provide preventive services and reduce the rate of caesarean sections. This study was designed and aimed to compare the rates of caesarean section and women's awareness and preference for mode of delivery before and after the implementation of the family physician program in health centres with and without an increases in the density of midwives.

## Materials and Methods

### Design and Setting

This cross-sectional study aimed to determine the effects of recruiting midwives into the family physician program on the rates of caesarean section, women's awareness and preference for mode of delivery. The rural health centres that implemented the family physician program in this study were divided into two groups: health centres that had an increase in their density of midwives during the study’s time frame and health centres that had no change in their density of midwives during the same period. The rates of caesarean section, women's awareness and preference for mode of delivery were compared between participants who had been referred to the rural health houses to vaccinate their two-month-old children belonging to one of these two groups.

### Data Collection and Variables

The unprocessed data that was used as our source of data prior to establishing the family physician program in 2005 was obtained from the Integrated Monitoring Evaluation System Survey (IMES). In 2005, a stratified, multistage probability cluster sampling method, with a probability in proportion to size procedure, had been used[28].

The sample size in each health house was calculated using the following formula:

$$\frac{Tmr\times N2}{\sum Tmr}=M$$

M = Total number of interviews in each health house

Tmr = Total number of vaccinations carried out for two-month-old children during the last recent month in each health house

∑Tmr = Total number of vaccinations carried out for two-month-old children during the last recent month in health houses selected from each district

N2 = Total number of samples in the specified rural area

Random systematic sampling had been used to sample two-month-old children in health house. The sampling frame was the list of childcare users which covered more than 99% of two-month-old children in rural area[28].

The researcher collected the data post to the implementation of family physician program in 2013. The sampling in 2013 was conducted in the same rural health houses with the same sample sizeand consistent with the sampling method which had been used for the IMES in 2005 (Table 1). All mothers who had been invited to participate in the study, accepted the invitation.

**Table 1:** Sampling frame in rural area of Kurdistan province in the survey of 2005 and 2013.

District	Health center(n)	Health house(n)	Recruited subjects(n)
Sanandaj	9	16	50
Kamyaran	12	33	114
Ghorveh	11	22	98
Marivan	7	18	82
Baneh	9	19	90
Saghez	13	33	76
Bijar	10	20	102
Divandareh	6	15	56
Total	77	176	668

The data collection tool for the study was questionnaire which had been used for the IMES in 2005. An expert committee of the Ministry of Health and Medical Education had tested this questionnaire for validity and reliability in the years 2002–2004[28].

#### Definition of variables

In this study, the percentage of mothers aware of the benefits of natural childbirth indicated the percentage of mothers who knew that natural childbirth was associated with at least three of the following outcomes: 1. Lower costs; 2. Less bleeding during childbirth; 3. Lower risk of infection; 4. More successful breastfeeding; 5. Shorter hospital stay and 6. Avoidance of anaesthetic-related risks.

The density of rural community health(Bhevarz) workers,family physicians or midwives was calculated as the total number of professionals per 1000 persons in the population. We used quintiles to summariz the frequency distribution of density of midwives, density of family physicians, and density of rural community health (Bhevarz) workers.

**Fig 1:**
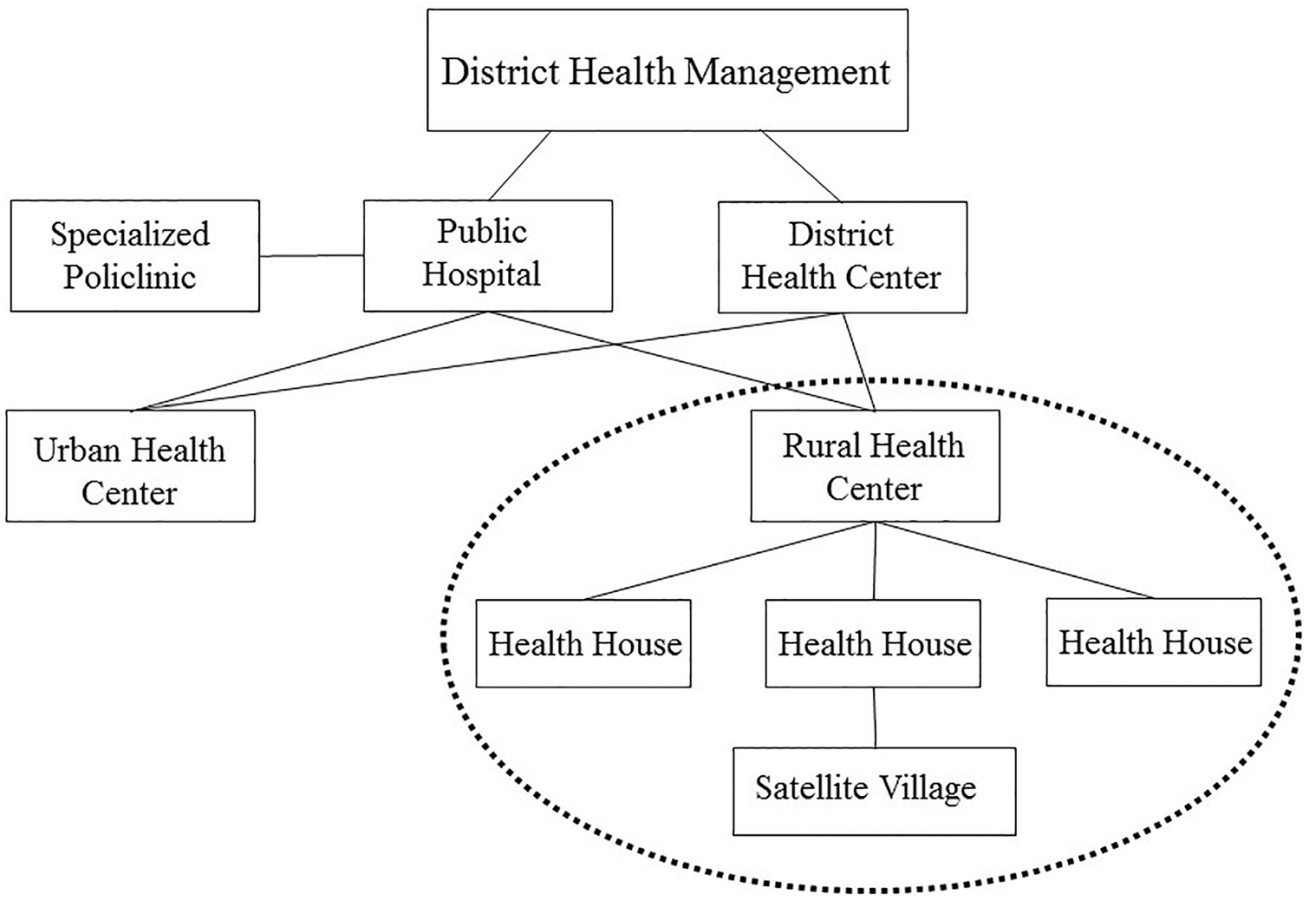
A simplified structure of PHC network in rural areas of Iran [29].

The rural health house is the most peripheral health delivery facility in rural areas and it is the place where behvarz (a formally trained community health worker) works. Each health house is designed to cover a target population of about 1500 people. The services provided by behvarzes in health house are supported and supervised by family physicians and midwives; there group of service providors are resident in in rural health centres (Fig 1).

Socioeconomic situation: The characteristics of the villages covered by the health centres were collected, including data on schools, electricity, pipedwater, gas pipelines, mailboxes, public Internet access and public transportation access and access to newspapers, banks and stores. These data were collected at both years of 2005 and 2013. These information were collapsed at the level of health centres and weighted based on the population of each village. This score was used to assess the socioeconomic status of the study setting using principal component analysis (PCA) resulting a Rho = 0.2630(the percentage of variance explained by the first factor), removal of variables with high unexpected percentages increased the Rho to 0.4023.

### Ethical Considerations

The study was approved by the Research Ethics Committee of the Shahid Beheshti University of Medical Sciences and written informed consent was obtained from the participants.

### Statistical Analysis

The data was analysed using R and Stata software to assess the effects of family physician programs on factors associated with caesarean section rates, various statistical models, including the difference-in-differences model, non-parametric and parametric combined models were explored, of which Matchit was the most appropriate model. Matchit model is optimum as propensity score matching is a powerful method for evaluating the community-based interventions and clarify the causal relations; it is used when the random implementation of an intervention is neither practical nor ethical. This method can help to reduce selection bias, which is common in observational studies [30, 31]. In our study, the intervention was not randomly applied to the health centres, so Matchit was used to show that the changes in indices associated with caesarean section rates before and after the implementation of family physician program had happened under equal conditions. For this reason, the Average Treatment Effect (ATE) was calculated.

The final form of difference in difference model was as follows:

$$Y_{icd}\;=\;\beta_{0}+\beta_{1}k_{d}+\beta_{2}T_{c}+\beta_{3}\left(k_{d}\text{*}T_{c}\right)+\beta_{4}\text{PM}+\beta_{i}\left(OHRD\right)+\beta_{1}\text{X}+\delta_{d}+{µ}_{c}+\varepsilon_{icd}$$

Y: index of caesarean section or awareness of the benefits of natural childbirth or preference for a caesarean section

T: 0 = 2005; 1 = 2013

K: 0 = health centers without increase in their density of midwives; 1 = health centers with increase in their density of midwives.

T * k: It was equal to 1 when the health centers have increase in their density of midwives in 2013.

*b*₃: mean changes in caesarean section or awareness of the benefits of natural childbirth or preference for a caesarean section index in 2013 compared with 2005 in the health centers with increase in their density of midwives compared with the health centers that did not.

***X****i*:confounding variables such as mother’s age, mother’s education, density of health workers (Behvarz), density of family physician, population of the village, Sex ratio, Socio-economic status etc were controlled in this study.

To neutralize the effects of randomly assignment to different districts, the districts of Kurdistan province were entered into the model as indicator variables.

## Results

Table 2 demonstrated the percentage of women with awareness and preference for mode of delivery and the percentage of women undergoing caesarean sections in 2005 and 2013 in the health centers with and without an increase in midwives density after the implementation of family physician program.

**Table 2:** Characteristics of the study population by Intervention in family physician program (2005: N = 668, 2013: N = 668).

		Intervention (increase in the density of midwives)in family physician program			
		increase in density		no increase in density	
Variable	Variable category	2005	2013	2005	2013
Age groups	<18 years	20(71.4%)	8(66.7%)	8(28.6%)	4(33.3%)
	18–35 years	470(81.3%)	447(80.4%)	108(18.7%)	109(19.6%)
	≥35 years	47(75.8%)	82(82%)	15(24.2%)	18(18%)
Job	Employed	497(79.4%)	486(80.9%)	129(20.6%)	115(19.1%)
	Unemployed	40(95.2%)	51(76.1%)	2(4.8%)	16(23.9%)
Education	Illiterate	194(77.6%)	85(80.2%)	56(22.4%)	21(19.8%)
	Literate	343(82.1%)	452(80.4%)	75(17.9%)	110(19.57%)
Parity	High risk (> = 5 pregnancy)	41(67.2%)	24(88.9%)	20(32.8%)	3(11.1%)
	Low risk (< 5 pregnancy)	496(81.7%)	513(80%)	111(18.3%)	128(20%)
Smoking or drug abuse	Yes	64(74.4%)	29(80.6%)	22(25.6%)	7(19.4%)
	No	473(81.3%)	508(80.4%)	109(18.7%)	124(19.6%)
History of medical disease or high risk obstetrical condition	Yes	86(81.1%)	70(85.4%)	20(18.9%)	12(14.6%)
	No	451(80.2%)	467(79.7%)	111(19.8%)	119(20.3%)
Prenatal complication	Yes	218(82.6%)	190(79.2%)	46(17.4%)	50(29.8%)
	No	319(79%)	347(81.1%)	85(21%)	81(18.9%)
Women’s awareness of the benefits of natural childbirth	Yes	228(85.1%)	327(78.8%)	40(14.9%)	88(21.2%)
	No	309(77.2%)	210(83%)	91(22.8%)	43(17%)
Women’s preference for caesarean section	Yes	82(86.3%)	63(75%)	13(13.7%)	21(25%)
	No	455(79.4%)	474(81.2%)	118(20.6%)	110(18.8%)
Cesarean section	Yes	113(77.4%)	181(80.4%)	33(22.6%)	44(19.6%)
	No	424(81.2%)	356(80.4%)	98(18.8%)	87(19.6%)

The percentage of health centers in which the percentage of midwives’ density was in the fifth quintile was 6.49% in 2005 and it increased to 33.77% in 2013 (Table 3).

**Table 3:** Density distribution of midwives, family physicians and Bhevarzes in health centers in the survey of 2005 and 2013.

Variable	Variable category	Year of study	
		2005	2013
Density of midwives	First quintile	29(37.66%)	1(1.3%)
	Second quintile	20 (25.97%)	11(14.29%)
	Third quintile	13(16.88%)	18(23.38%)
	Fourth quintile	10(12.99%)	21(27.27%)
	Fifth quintile	5(6.49%)	26(33.77%)
Density of family physicians	First quintile	27(35.06%)	3(3.90%)
	Second quintile	16(20.78%)	15(19.48%)
	Third quintile	13(16.88%)	18(23.38%)
	Fourth quintile	12(15.58%)	19(24.68%)
	Fifth quintile	9(11.69%)	22(28.57%)
Density of rural community health (Bhevarz) workers	First quintile	19 (24.68%)	11(14.29%)
	Second quintile	19(24.68%)	12(15.58%)
	Third quintile	15(19.48%)	16(20.78%)
	Fourth quintile	14(18.8%)	17(22.08%)
	Fifth quintile	10(12.99%)	21(27.27%)

Using difference-in-differences model (Table 4), the resutls showed no significant change in the participants’awareness of the benefits of natural childbirth between 2005 and 2013 in health centres where there was an increase in the density of midwives, compared with those health centres that showed no increase,OR = 0.73, 95% CI:(0.47–1.13),P = 0.16. Moreover, the results showed that awareness of the benefits of natural childbirth had increased three-fold between 2005 and 2013(OR = 3, 95% CI: (1.95–4.62),p>0.001).

**Table 4:** Difference in difference modeling on women’s awareness of the benefits of natural childbirth in rural areas of Kurdistan province.

		Women’s awareness of the benefits of natural childbirth (yes, no)
		Difference in differences model
Variable	Variable category	OR(CI)
Interaction between intervention and time	1	0.73(0.47–1.13)P = 0.16
	0	
Year	2013	3(1.95–4.62)p>0.001
	2005	
Intervention (increase in the density of midwives)	yes	1.5(0.99–2.22)P = 0.051
	No	
Age	Continuous quantitative variable	1.01(0.99–1.04)P = 0.24
Parity	Continuous quantitative variable	0.93(0.83–1.04)P = 0.23
Education	Illiterate	1.2(0.9–1.61)P = 0.19
	Literate	
Job	Employed	1.2(0.77–1.77)P = 0.43
	Unemployed	
Smoking or drug abuse	yes	1(0.68–1.48)P = 0.98
	No	
Density of family physician staffs	Continuous quantitative variable	0.7(0.22–2.12)P = 0.51
Density of rural community health (Bhevarz) workers	Continuous quantitative variable	0.42(0.06–3.04)P = 0.39
Socio-economic features	Continuous quantitative variable	0.99(0.95–1.03)P = 0.75
Logarithm of the rural population	Continuous quantitative variable	0.68(0.43–1.1)P = 0.1
Sex ratio	Continuous quantitative variable	1(0.97–1.02)P = 0.95

The difference-in-differences model(Table 5) for the intervention showed that there was no significant change in the preference for caesarean sections between 2005 and 2013 in health centres that showed an increase in the density of midwives, compared with health centres that had no increase, (OR = 0.47, 95% CI:(0.17–1.3),P = 0.13). And the results showed that a preference for caesarean sections had decreased by 46% between 2005 and 2013, (OR = 0.54, 95% CI:(0.32–0.9), P = 0. 001). In particular, this preference decreased by 41% among women who were aware of the benefits of natural childbirth(OR = 0.59, 95% CI:(0.22–0.85),P>0.001.

**Table 5:** Difference in difference modeling on women’s preference for caesarean section and undergoing caesarean section in rural areas of Kurdistan province.

		Women’s preference for caesarean section (yes, no)	Undergoing caesarean section (yes, no)
		Difference in difference model	Difference in difference model
Variable	Variable category	OR(CI)	OR(CI)
Interaction between intervention and time	1	0.47(0.17–1.3)P = 0.13	1.38(0.71–2.7)P = 0.34
	0		
Year	2013	0.54(0.32–0.9)P = 0.001	2.04(1.22–3.41)P = 0.006
	2005		
Intervention (increase in the density of midwives)	yes	1.32(0.61–2.9)P = 0.48	0.91(0.54–1.52)P = 0.72
	No		
Age	Continuous quantitative variable	1(0.96–1.05)P = 0.72	0.99(0.97–1.02)P = 0.86
Parity	Continuous quantitative variable	0.94(0.79–1.13)P = 0.53	1.03(0.91–1.17)P = 0.63
Education	Illiterate	1.43(0.88–2.32)P = 0.14	1.14(0.82–1.59)P = 0.42
	Literate		
Job	Employed	0.92(0.49–1.71)P = 0.79	0.82(0.52–1.32)P = 0.43
	Unemployed		
Smoking or drug abuse	yes	0.89(0.47–1.67)P = 0.72	1.14(0.74–1.75)P = 0.55
	No		
Density of family physician staffs	Continuous quantitative variable	2.8(0.44–4.45)P = 0.26	0.55(0.14–2.1)P = 0.38
Density of rural community health (Bhevarz) workers	Continuous quantitative variable	0.54(0.03–1.03)P = 0.69	0.95(0.12–3.4)P = 0.96
Socio-economic features	Continuous quantitative variable	1(0.95–1.07)P = 0.76	0.97(0.93–1.02)P = 0.28
Logarithm of the rural population	Continuous quantitative variable	1.61(0.96–2.96)P = 0.07	0.92(0.64–1.32)P = 0.68
Sex ratio	Continuous quantitative variable	0.97(0.94–1.01)P = 0.25	0.97(0.94–1.01)P = 0.13
Women’s awareness of the benefits of natural childbirth	yes	0.59(0.22–0.85)P>0.001	0.86(0.65–1.13)P = 0.28
	No		
Pregnancy complications	yes	0.7(0.49–1.02)P = 0.07	1.6(1.2–2.05)P>0.0001
	No		
History of medical or obstetric high risk conditions	yes	0.71(0.41–1.24)P = 0.24	1.26(0.89–1.8)P = 0.18
	No		
Natural childbirth	yes	0.79(0.54–1.2)P = 0.25	
	No		
Women’s preference for caesarean section	yes		1.22(0.82–1.8)P = 0.31
	No		

The difference-in-differences model (Table 5)showed no significant change in the caesarean section rates between 2005 and 2013 for health centres that showed an increase in the density of midwives, compared with the health centres without any increase,OR = 1.38(0.71–2.7),P = 0.34, And the results demonstrated two times increase in caesarean section between 2005 and 2013(OR = 2.04, 95%CI(1.22–3.41), P = 0.006. This odds ratio increased by 60% among women suffering from pregnancy complicationsOR = 1.6(1.2–2.05)P>0.001.

Based on the Matchit model analysis, The Matchit model showed a significant mean increase (14%, 95% CI(0.03–0.25) in women’s awareness of the benefits of natural childbirth between 2005 and 2013 in health centres where the density of midwives increased compared with health centres without such an increase.(Table 6).

**Table 6:** The estimated effect on increasing inthe density of midwives in family physician program according to Matchit model on caesarean section, women’s preference for caesarean section, and women’s awareness of the benefits of natural childbirth in the rural health centers in Kurdistan province.

	Matchit		
		95% Confidence Interval	
Variable	Average effect	Low	High
Women’s awareness of the benefits of natural childbirth	0.14	0.03	0.25
Women’s preference for caesarean section	0.02	-0.03	0.05
Undergoing caesarean section	-0.02	-0.13	0.07

## Discussion

The results of this study showed that an increase in the density of midwives in the family physician program led to an increased women's awareness of the benefits of natural childbirth among mothers from 2005 to 2013 in health centres where there was an increase in the density of midwives, compared with those health centres without an increase. Women's awareness of the benefits of natural childbirth was also found to have an impact on their preference for delivery by caesarean section. On the other hand, the density of midwives had no significant effect on the caesarean section rates or preference for caesarean section delivery.

Studies conducted in Finland and Norway showed that proper consultation together with support from health care providers was associated with lower rates of mothers requesting delivery by caesarean section[32, 33]. These studies highlight the important role of health care providers in influencing delivery choices among women[34].

It seems that midwives working in the family physician program in the present study had a positive attitude toward natural childbirth, which contributed to increased awareness of its benefits. This relationship indicates that the attitudes of health care providers toward caesarean sections play an influential role in women’s awareness of the benefits of natural childbirth and their choices in regard to it[10, 35].

In this study, women's awareness of the benefits of natural childbirth was associated with a lower likelihood of delivery via caesarean section; however, the decline in the rate of caesarean sections was not significant. Conversely, a high preference for caesarean sections was associated with an increase in caesarean section rates, but this increase was also not significant. Other research, such as a study conducted in a public hospital in Rio de Janeiro, Brazil, has shown a similar relationship between a higher preference for caesarean section delivery and increases in the rates of caesarean sections[16].

The results of this study showed that the preference for delivery by caesarean section was not the main factor behind increasing caesarean section rates, which parallels the finding of a systematic review on the increasing caesarean section rates[36]. Cochrane’s review showed that training mothers in natural childbirth skills had no effect on delivery rates involving natural childbirth[37].

Little evidence exists on the impact of prenatal group classes during pregnancy, such as classes, which train mothers in relaxation techniques and the benefits of natural childbirth, on women’s delivery choices, and they have not increased the rate of natural childbirth [38]. In addition, evidence suggests that training individuals about the benefits of avoiding caesarean sections does not increase the rate of natural childbirth[26, 38]. Generally, however, the effects of prenatal education on natural childbirth remain unknown [38]. In nulliparous women without medical indications for caesarean section, the recommendations offered by gynaecologists or obstetrics can be a strong predictor of delivery choice[33]. The results of an observational study on prenatal care showed that many women tend to follow the suggestions of physicians and specialists when making decisions about having a caesarean section[39].

In Iran, part of the increase in the caesarean section rates is due to health care providers’ concerns about legal issues that may occur during natural childbirth; to avoid engaging in legal issues, obstetricians and midwives prefer delivering infants by caesarean section[12]. Therefore, in this study, the chance of undergoing caesarean section delivery was 60% higher for mothers who were suffering from pregnancy complications. Although some low risk pregnancies with a perfect prenatal care may end with a major complication during labour, needing an emergent caesarean section, but it is not a common feature and the majority of pregnancy complications can be properly managed by early diagnosis. It seems that early detection of pregnancy complications by midwives can be achieved through following integrated maternal health care programs and early referral of mothers to the health care system, because many indications for caesarean section are related to medical issues[40, 41]. A systematic review conducted in this field showed that when women did not have any complications in their current or previous pregnancies, they rarely asked for a caesarean section [10]. It is remarkable that caesarean sections are not the only solution for controlling all of these complications. Therefore, the World Health Organization has reported that only about 15% of deliveries in any region of the world meet the indications for caesarean section [8, 9].

On the other hand, it seems that the attitude of obstetricians and gynaecologists may be the most important factor influencing the rate of caesarean sections[42]. Obstetricians prefer to spend less time on delivery processes; financial issues and insurance payments also influence their choices[12]. However, these factors are often not clearly stated by obstetricians[12].

The complex relationships between parents, community, health care providers and cultural, social and economic factors have all contributed to increases in caesarean section rates in different countries; the increase in the caesarean section rate is sometimes primarily due to women’s preferences for caesarean sections without a medical need, and sometimes it is due to recommendations offered by gynaecologists[19]. Therefore, it seems that an increase in the density of midwives in the family physician program had no significant effect on caesarean section rates, making it necessary to use various strategies to reduce this rate.

Overall, several studies have examined the associations between human resources and health indicators and have found different results; some have reported positive associations between these constructs, while others have reported negative association[43–50]. The results of these previous studies are influenced by various factors. For instance, many of these studies did not assess the effects of health worker density on health indicators at the individual level, but rather, they have calculated health indicators at the level of district or province or country. The relationship between the variables in a district may not reflect the relationship between variables at the individual level. These studies also did not control for socioeconomic status at the individual or family level; instead, they usually used variables such as the average level of education within a district, although the socioeconomic statuses of individuals are a preferred measure that can increase the precision of the study. Nevertheless, previous studies have shown that the measurement of cumulative socioeconomic variables is valid at the district level[51]. In many of these studies, there is no data about the actual use of family physician services by each individual[52]. One of the strengths of the present study is that the data was collected at an individual level; another is the use of Matchit statistical model. A third strength of this study was that it compared the services received by mothers with pre-defined standards proposed by the Ministry of Health and Medical Education, made an assessment of the services, and evaluated the consistency between the services provided and the standard services. It showed how closely service providers are following the standards when providing primary health care services. The study was limited by the fact that we only had two time points for data collection, in 2005 and 2013, and therefore we were not able to show changes occurring during the years between 2005 and 2013. Another limitation is the result of the present study may not be extrapolated to large cities.

## Conclusions

Based on the Matchit model analysis, the increase in the density of midwives in the family physician program contributed to increased participant awareness of the benefits of natural childbirth. Based on the difference in difference model analysis, the study also found that women’s awareness of the benefits of natural childbirth negatively influenced their preference for delivery by caesarean section, but this relationship did not have a significant impact on actual caesarean section rates.

The results showed that the interventions to reduce caesarean section rates by educating patients about risk factors associated with each type of delivery method is not enough, because the complex relationships between parents, community, health care providers and cultural, social and economic factors may have a larger influence on caesarean section rates in different countries. Therefore, it is necessary to use more diverse strategies to reduce the caesarean section rates such as integrated electronic medical records for pregnant women from the beginning of pregnancy until delivery should be created.

## Supporting Information

S1 Table 20% of original data. Characteristics of the study populationin rural areas of Kurdistan and individual data.
(DOC)
