# Supplementary material for: Effects of Recruiting Midwives into a Family Physician Program on Women's Awareness and Preference for Mode of Delivery and Caesarean Section Rates in Rural Areas of Kurdistan
Source: PLoS One. 2016 Apr 8;11(4):e0151268. doi: 10.1371/journal.pone.0151268 (PMC4825997; doi:10.1371/journal.pone.0151268)
Supplement: S1 Table — Characteristics of the study populationin rural areas of Kurdistan and individual data. (DOC) [file pone.0151268.s001.doc]

**S1 Table. 20% of Original Data.** Characteristics of the study population in rural areas of Kurdistan and individual data

| year | Health center | sr | district | Age | Education | Parity | Job | | smoking or drug abuse | history of complication in pregnancy | history medical disease in Pregnancy | History of medical or obstetric high risk conditions | complication in this pregnancy | Women’s awareness of the benefits of natural childbirth | Women’s preference for caesarean section | Cesarean section | Intervention (increasein thedensity of midwives) | Interaction between intervention and time | Density of family physicians | Density of Midwives | Density of rural community health (Bhevarz) workers | Logarithm of the rural population | Sex ratio | Socio-economic status |
| --- | --- | --- | --- | --- | --- | --- | --- | --- | --- | --- | --- | --- | --- | --- | --- | --- | --- | --- | --- | --- | --- | --- | --- | --- |
| 0 | 1 | 90 | 2 | 27 | 1 | 3 | | 4 | 0 | 0 | 0 | 0 | 0 | 1 | 1 | 0 | 1 | 0 | 0.0123686 | 0.0123686 | 0.148423 | 8.997766 | 102.479 | 9.035476 |
| 1 | 66 | 33 | 8 | 30 | 1 | 2 | | 1 | 1 | 0 | 0 | 0 | 0 | 1 | 0 | 0 | 1 | 1 | 0.0225428 | 0.0225428 | 0.1803426 | 8.397509 | 103.114 | -0.4675424 |
| 0 | 18 | 73 | 1 | 20 | 1 | 1 | | 4 | 1 | 1 | 0 | 1 | 0 | 0 | 0 | 1 | 1 | 0 | 0 | 0 | 0.094038 | 8.578665 | 102.707 | 2.15365 |
| 1 | 3 | 30 | 6 | 30 | 1 | 2 | | 4 | 0 | 0 | 0 | 0 | 0 | 0 | 0 | 0 | 1 | 1 | 0.0499251 | 0.0499251 | 0.5242137 | 8.295548 | 104.806 | 1.110284 |
| 0 | 34 | 99 | 2 | 36 | 0 | 7 | | 4 | 0 | 0 | 0 | 0 | 0 | 0 | 0 | 0 | 0 | 0 | 0.0329443 | 0.0329443 | 0.1506024 | 7.884576 | 101.977 | 2.049861 |
| 0 | 1 | 24 | 2 | 26 | 1 | 1 | | 4 | 1 | 1 | 0 | 1 | 0 | 1 | 1 | 0 | 1 | 0 | 0.0123686 | 0.0123686 | 0.148423 | 8.997766 | 102.479 | 9.035476 |
| 1 | 15 | 110 | 5 | 30 | 1 | 3 | | 4 | 0 | 0 | 0 | 0 | 0 | 1 | 0 | 0 | 1 | 1 | 0.0174201 | 0.0174201 | 0.0958105 | 9.348449 | 108.064 | 2.942514 |
| 0 | 61 | 92 | 5 | 29 | 0 | 4 | | 4 | 0 | 0 | 0 | 0 | 0 | 1 | 0 | 0 | 1 | 0 | 0 | 0 | 0.1015572 | 7.990915 | 114.68 | -0.9706272 |
| 0 | 10 | 13 | 8 | 34 | 1 | 2 | | 2 | 0 | 0 | 0 | 0 | 0 | 1 | 0 | 0 | 1 | 0 | 0 | 0 | 0.3475843 | 8.657651 | 100.978 | 4.175152 |
| 1 | 45 | 47 | 4 | 22 | 1 | 1 | | 1 | 0 | 1 | 0 | 1 | 1 | 0 | 0 | 0 | 1 | 1 | 0.0294774 | 0.0446628 | 0.3573024 | 7.713785 | 107.123 | 14.63733 |
| 0 | 47 | 64 | 4 | 28 | 1 | 2 | | 4 | 0 | 1 | 0 | 1 | 1 | 0 | 0 | 1 | 0 | 0 | 0.0194894 | 0.0194894 | 0.0779575 | 8.543056 | 108.408 | 2.371052 |
| 0 | 10 | 19 | 8 | 26 | 1 | 2 | | 4 | 0 | 0 | 0 | 0 | 0 | 1 | 0 | 0 | 1 | 0 | 0 | 0 | 0.3475843 | 8.657651 | 100.978 | 4.175152 |
| 0 | 10 | 9 | 8 | 36 | 1 | 6 | | 4 | 0 | 0 | 0 | 0 | 0 | 0 | 0 | 0 | 1 | 0 | 0 | 0 | 0.3475843 | 8.657651 | 100.978 | 4.175152 |
| 0 | 7 | 34 | 7 | 26 | 0 | 2 | | 4 | 1 | 0 | 0 | 0 | 0 | 0 | 0 | 0 | 1 | 0 | 0.0109123 | 0.0095482 | 0.0654736 | 9.123038 | 107.143 | 2.840596 |
| 1 | 14 | 80 | 5 | 40 | 0 | 5 | | 4 | 0 | 0 | 0 | 0 | 1 | 1 | 0 | 0 | 1 | 1 | 0.0403986 | 0.0269324 | 0.1481282 | 8.912743 | 106.45 | 6.199605 |
| 0 | 5 | 12 | 7 | 19 | 1 | 1 | | 4 | 0 | 1 | 0 | 1 | 1 | 0 | 0 | 0 | 1 | 0 | 0.0127559 | 0.006378 | 0.0574016 | 9.660077 | 104.553 | 10.75591 |
| 0 | 3 | 48 | 6 | 23 | 1 | 1 | | 4 | 1 | 1 | 0 | 1 | 0 | 1 | 1 | 0 | 1 | 0 | 0.0105731 | 0.0105731 | 0.2114612 | 9.154616 | 141.399 | -0.8191268 |
| 0 | 72 | 32 | 6 | 29 | 1 | 2 | | 4 | 0 | 0 | 0 | 0 | 0 | 0 | 0 | 0 | 1 | 0 | 0.0126093 | 0.0105077 | 0.2017485 | 8.69081 | 104.258 | 1.426343 |
| 1 | 34 | 100 | 2 | 23 | 1 | 2 | | 1 | 0 | 0 | 0 | 0 | 1 | 1 | 0 | 0 | 0 | 0 | 0.0426257 | 0.0426257 | 0.2557545 | 7.760467 | 104.534 | 1.028819 |
| 0 | 41 | 1 | 3 | 26 | 1 | 3 | | 4 | 0 | 0 | 0 | 0 | 0 | 1 | 0 | 0 | 1 | 0 | 0.022259 | 0.022259 | 0.1780717 | 8.276649 | 105.596 | -0.4681446 |
| 0 | 13 | 41 | 5 | 37 | 0 | 10 | | 4 | 0 | 0 | 0 | 0 | 0 | 1 | 0 | 0 | 1 | 0 | 0.0125881 | 0.0125881 | 0.0629406 | 8.980172 | 107.849 | 4.278709 |
| 0 | 30 | 70 | 2 | 22 | 1 | 2 | | 4 |  |  | 0 | 0 | 1 | 0 | 0 | 1 | 1 | 0 | 0.0201242 | 0.0201242 | 0.1609936 | 8.377471 | 101.296 | 9.05479 |
| year | Health center | sr | district | Age | Education | Parity | Job | | smoking or drug abuse | history of complication in pregnancy | history medical disease in Pregnancy | History of medical or obstetric high risk conditions | complication in this pregnancy | Women’s awareness of the benefits of natural childbirth | Women’s preference for caesarean section | Cesarean section | Intervention (increasein thedensity of midwives) | Interaction between intervention and time | Density of family physicians | Density of Midwives | Density of rural community health (Bhevarz) workers | Logarithm of the rural population | Sex ratio | Socio-economic status |
| 1 | 64 | 62 | 8 | 18 | 1 | 1 | 4 | | 0 | 1 | 0 | 1 | 0 | 0 | 0 | 0 | 1 | 1 | 0.0090501 | 0.0217202 | 0.2606429 | 8.434681 | 109.7 | -0.5144128 |
| 0 | 54 | 83 | 5 | 32 | 0 | 5 | 4 | | 0 | 0 | 0 | 0 | 1 | 1 | 1 | 0 | 1 | 0 | 0.0153799 | 0.0096124 | 0.1076592 | 8.779865 | 114.234 | -0.2427101 |
| 0 | 3 | 30 | 6 | 30 | 0 | 2 | 4 | | 0 | 0 | 0 | 0 | 0 | 1 | 1 | 1 | 1 | 0 | 0.0105731 | 0.0105731 | 0.2114612 | 9.154616 | 141.399 | -0.8191268 |
| 0 | 37 | 61 | 2 | 26 | 1 | 3 | 4 | | 1 | 1 | 0 | 1 | 1 | 1 | 0 | 0 | 1 | 0 | 0.0186335 | 0.0186335 | 0.2236025 | 8.300281 | 104.211 | 0.5670744 |
| 1 | 14 | 35 | 5 | 27 | 1 | 2 | 4 | | 0 | 0 | 0 | 0 | 0 | 0 | 0 | 0 | 1 | 1 | 0.0403986 | 0.0269324 | 0.1481282 | 8.912743 | 106.45 | 6.199605 |
| 0 | 31 | 31 | 2 | 28 | 1 | 3 | 4 | | 0 | 0 | 0 | 0 | 0 | 0 | 0 | 0 | 1 | 0 | 0.0221239 | 0.0221239 | 0.2528445 | 8.282736 | 94.7317 | 1.680652 |
| 1 | 60 | 55 | 5 | 38 | 1 | 3 | 4 | | 0 | 0 | 0 | 0 | 0 | 1 | 0 | 0 | 1 | 1 | 0.0206313 | 0.0206313 | 0.0825253 | 8.486115 | 109.012 | 1.135782 |
| 1 | 17 | 88 | 1 | 39 | 0 | 5 | 4 | | 0 | 1 | 0 | 1 | 1 | 0 | 0 | 0 | 1 | 1 | 0.0521921 | 0.026096 | 0.3131524 | 8.251143 | 104.92 | 5.032202 |
| 1 | 48 | 32 | 4 | 21 | 1 | 1 | 4 | | 0 | 1 | 0 | 1 | 0 | 1 | 0 | 1 | 1 | 1 | 0.0291375 | 0.0291375 | 0.1748252 | 8.140899 | 104.651 | 2.82518 |
| 0 | 51 | 51 | 4 | 22 | 1 | 1 | 4 | | 0 | 1 | 0 | 1 | 1 | 1 | 0 | 0 | 1 | 0 | 0.0140371 | 0.0140371 | 0.1965188 | 8.178078 | 107.939 | -0.5497003 |
| 0 | 79 | 74 | 6 | 29 | 1 | 3 | 4 | | 0 | 1 | 0 | 1 | 0 | 1 | 0 | 1 | 1 | 0 | 0.0218477 | 0.0218477 | 0.1747815 | 8.295299 | 104.337 | 1.275381 |
| 1 | 51 | 50 | 4 | 29 | 1 | 2 | 4 | | 0 | 0 | 0 | 0 | 1 | 0 | 0 | 0 | 1 | 1 | 0.0295103 | 0.0322165 | 0.2255155 | 8.040447 | 110.583 | 0.1859285 |
| 1 | 36 | 58 | 2 | 26 | 1 | 1 | 4 | | 0 | 1 | 0 | 1 | 1 | 0 | 0 | 0 | 1 | 1 | 0.0279744 | 0.0337041 | 0.2359286 | 7.995306 | 101.289 | -0.127527 |
| 1 | 66 | 39 | 8 | 34 | 0 | 3 | 4 | | 0 | 0 | 0 | 0 | 0 | 0 | 0 | 0 | 1 | 1 | 0.0225428 | 0.0225428 | 0.1803426 | 8.397509 | 103.114 | -0.4675424 |
| 1 | 37 | 62 | 2 | 21 | 1 | 2 | 4 | | 0 | 0 | 0 | 0 | 0 | 1 | 1 | 0 | 1 | 1 | 0.029274 | 0.029274 | 0.263466 | 8.136226 | 104.796 | 0.399502 |
| 1 | 10 | 25 | 8 | 21 | 1 | 1 | 4 | | 0 | 1 | 0 | 1 | 0 | 1 | 0 | 1 | 1 | 1 | 0.0099835 | 0.0149753 | 0.1198023 | 9.905136 | 106.164 | 1.411475 |
| 0 | 45 | 67 | 4 | 30 | 1 | 2 | 4 | | 0 | 0 | 0 | 0 | 0 | 1 | 0 | 0 | 1 | 0 | 0.0223547 | 0.0223547 | 0.2384501 | 8.118207 | 107.997 | 0.1683084 |
| 0 | 3 | 72 | 6 | 26 | 1 | 4 | 4 | | 0 | 0 | 0 | 0 | 0 | 1 | 0 | 0 | 1 | 0 | 0.0105731 | 0.0105731 | 0.2114612 | 9.154616 | 141.399 | -0.8191268 |
| 0 | 45 | 82 | 4 | 16 | 1 | 2 | 4 | | 0 | 0 | 0 | 0 | 0 | 1 | 0 | 0 | 1 | 0 | 0.0223547 | 0.0223547 | 0.2384501 | 8.118207 | 107.997 | 0.1683084 |
| 0 | 17 | 6 | 1 | 28 | 0 | 2 | 4 | | 0 | 0 | 0 | 0 | 0 | 0 | 0 | 0 | 1 | 0 | 0.0182242 | 0.0145794 | 0.1166351 | 8.833317 | 105.359 | 1.309201 |
| 1 | 17 | 85 | 1 | 30 | 0 | 4 | 4 | | 0 | 0 | 0 | 0 | 0 | 1 | 0 | 0 | 1 | 1 | 0.0521921 | 0.026096 | 0.3131524 | 8.251143 | 104.92 | 5.032202 |
| 0 | 30 | 30 | 2 | 26 | 1 | 1 | 4 | | 0 | 1 | 0 | 1 | 1 | 0 | 0 | 0 | 1 | 0 | 0.0201242 | 0.0201242 | 0.1609936 | 8.377471 | 101.296 | 9.05479 |

| year | Health center | sr | district | Age | Education | Parity | Job | smoking or drug abuse | history of complication in pregnancy | history medical disease in Pregnancy | History of medical or obstetric high risk conditions | complication in this pregnancy | Women’s awareness of the benefits of natural childbirth | Women’s preference for caesarean section | Cesarean section | Intervention (increasein thedensity of midwives) | Interaction between intervention and time | Density of family physicians | Density of Midwives | Density of rural community health (Bhevarz) workers | Logarithm of the rural population | Sex ratio | Socio-economic status |
| --- | --- | --- | --- | --- | --- | --- | --- | --- | --- | --- | --- | --- | --- | --- | --- | --- | --- | --- | --- | --- | --- | --- | --- |
| 0 | 52 | 31 | 4 | 22 | 1 | 1 | 4 | 0 | 1 | 0 | 1 | 1 | 0 | 0 | 1 | 0 | 0 | 0.0267502 | 0.0133751 | 0.1681443 | 8.785998 | 109.076 | 5.935247 |
| 0 | 45 | 69 | 4 | 33 | 0 | 4 | 4 | 0 | 0 | 0 | 0 | 1 | 0 | 0 | 0 | 1 | 0 | 0.0223547 | 0.0223547 | 0.2384501 | 8.118207 | 107.997 | 0.1683084 |
| 0 | 1 | 22 | 2 | 20 | 1 | 1 | 4 | 1 | 1 | 0 | 1 | 0 | 0 | 0 | 1 | 1 | 0 | 0.0123686 | 0.0123686 | 0.148423 | 8.997766 | 102.479 | 9.035476 |
| 1 | 34 | 96 | 2 | 23 | 1 | 1 | 4 | 0 | 1 | 1 | 2 | 1 | 0 | 0 | 0 | 0 | 0 | 0.0426257 | 0.0426257 | 0.2557545 | 7.760467 | 104.534 | 1.028819 |
| 1 | 10 | 21 | 8 | 22 | 1 | 1 | 4 | 0 | 1 | 0 | 1 | 0 | 0 | 0 | 0 | 1 | 1 | 0.0099835 | 0.0149753 | 0.1198023 | 9.905136 | 106.164 | 1.411475 |
| 0 | 1 | 19 | 2 | 37 | 1 | 3 | 4 | 0 | 0 | 0 | 0 | 0 | 1 | 0 | 0 | 1 | 0 | 0.0123686 | 0.0123686 | 0.148423 | 8.997766 | 102.479 | 9.035476 |
| 1 | 5 | 18 | 7 | 25 | 1 | 1 | 4 | 0 | 1 | 0 | 1 | 0 | 1 | 0 | 0 | 1 | 1 | 0.0176046 | 0.0117364 | 0.0528138 | 9.743378 | 101.549 | 10.75591 |
| 0 | 1 | 87 | 2 | 34 | 1 | 3 | 1 | 0 | 0 | 0 | 0 | 1 | 1 | 0 | 0 | 1 | 0 | 0.0123686 | 0.0123686 | 0.148423 | 8.997766 | 102.479 | 9.035476 |
| 0 | 10 | 23 | 8 | 21 | 0 | 2 | 4 | 0 | 0 | 1 | 1 | 1 | 0 | 0 | 0 | 1 | 0 | 0 | 0 | 0.3475843 | 8.657651 | 100.978 | 4.175152 |
| 1 | 14 | 30 | 5 | 22 | 1 | 1 | 4 | 0 | 1 | 0 | 1 | 0 | 0 | 0 | 0 | 1 | 1 | 0.0403986 | 0.0269324 | 0.1481282 | 8.912743 | 106.45 | 6.199605 |
| 1 | 47 | 58 | 4 | 23 | 1 | 2 | 4 | 0 | 0 | 0 | 0 | 1 | 1 | 0 | 0 | 0 | 0 | 0.0192752 | 0.0192752 | 0.1349268 | 8.554104 | 109.362 | 6.731958 |
| 1 | 7 | 41 | 7 | 29 | 1 | 3 | 4 | 0 | 0 | 0 | 0 | 0 | 0 | 0 | 0 | 1 | 1 | 0.0241633 | 0.0120817 | 0.108735 | 9.021235 | 105.589 | 3.677327 |
| 0 | 67 | 63 | 8 | 18 | 1 | 1 | 4 | 0 | 1 | 0 | 1 | 0 | 0 | 0 | 0 | 0 | 0 | 0.0122381 | 0.0122381 | 0.1370668 | 8.538367 | 106.427 | -0.6212724 |
| 1 | 32 | 35 | 2 | 38 | 1 | 1 | 4 | 0 | 1 | 0 | 1 | 0 | 1 | 0 | 0 | 1 | 1 | 0.0364034 | 0.0364034 | 0.2548234 | 7.918265 | 97.9107 | 0.1336359 |
| 1 | 45 | 45 | 4 | 37 | 1 | 5 | 4 | 0 | 0 | 0 | 0 | 0 | 0 | 0 | 0 | 1 | 1 | 0.0294774 | 0.0446628 | 0.3573024 | 7.713785 | 107.123 | 14.63733 |
| 0 | 14 | 64 | 5 | 27 | 0 | 1 | 4 | 0 | 1 | 0 | 1 | 0 | 1 | 0 | 0 | 1 | 0 | 0.0178015 | 0.0089008 | 0.1424121 | 9.326789 | 119.992 | 6.199605 |
| 1 | 42 | 53 | 3 | 38 | 0 | 6 | 4 | 0 | 0 | 0 | 0 | 1 | 1 | 0 | 1 | 1 | 1 | 0.022298 | 0.0243427 | 0.1217137 | 8.320691 | 106.225 | 0.3884559 |
| 0 | 10 | 11 | 8 | 28 | 1 | 2 | 4 | 0 | 0 | 0 | 0 | 1 | 1 | 0 | 1 | 1 | 0 | 0 | 0 | 0.3475843 | 8.657651 | 100.978 | 4.175152 |
| 1 | 17 | 83 | 1 | 25 | 1 | 2 | 4 | 0 | 1 | 0 | 1 | 0 | 1 | 0 | 0 | 1 | 1 | 0.0521921 | 0.026096 | 0.3131524 | 8.251143 | 104.92 | 5.032202 |
| 0 | 44 | 5 | 4 | 21 | 1 | 1 | 4 | 0 | 1 | 0 | 1 | 1 | 0 | 0 | 0 | 1 | 0 | 0.0080678 | 0.0080678 | 0.0968132 | 9.425049 | 109.4 | 11.53765 |
| 0 | 51 | 49 | 4 | 24 | 1 | 2 | 4 | 0 | 0 | 0 | 0 | 0 | 0 | 0 | 0 | 1 | 0 | 0.0140371 | 0.0140371 | 0.1965188 | 8.178078 | 107.939 | -0.5497003 |
| 0 | 77 | 1 | 6 | 33 | 1 | 5 | 4 | 1 | 0 | 0 | 0 | 1 | 0 | 0 | 0 | 1 | 0 | 0.0073202 | 0.0073202 | 0.1639728 | 9.052282 | 110.7 | 1.235519 |

| year | Health center | sr | district | Age | Education | Parity | Job | smoking or drug abuse | history of complication in pregnancy | history medical disease in Pregnancy | History of medical or obstetric high risk conditions | complication in this pregnancy | Women’s awareness of the benefits of natural childbirth | Women’s preference for caesarean section | Cesarean section | Intervention (increasein thedensity of midwives) | Interaction between intervention and time | Density of family physicians | Density of Midwives | Density of rural community health (Bhevarz) workers | Logarithm of the rural population | Sex ratio | Socio-economic status |
| --- | --- | --- | --- | --- | --- | --- | --- | --- | --- | --- | --- | --- | --- | --- | --- | --- | --- | --- | --- | --- | --- | --- | --- |
| 0 | 12 | 7 | 3 | 30 | 0 | 3 | 4 | 0 | 0 | 0 | 0 | 1 | 0 | 0 | 0 | 1 | 0 | 0.0110035 | 0.0110035 | 0.1320423 | 9.11471 | 112.188 | -0.0376237 |
| 1 | 38 | 35 | 3 | 20 | 1 | 1 | 4 | 0 | 1 | 0 | 1 | 0 | 1 | 0 | 1 | 1 | 1 | 0.015569 | 0.015569 | 0.1556905 | 8.76764 | 108.269 | 1.233363 |
| 0 | 32 | 36 | 2 | 40 | 1 | 4 | 4 | 0 | 0 | 0 | 0 | 1 | 1 | 0 | 0 | 1 | 0 | 0.0304878 | 0.0304878 | 0.3484321 | 7.962067 | 97.931 | -0.0168508 |
| 1 | 61 | 90 | 5 | 24 | 1 | 1 | 4 | 0 | 1 | 0 | 1 | 0 | 0 | 0 | 0 | 1 | 1 | 0.0343844 | 0.0375375 | 0.1501502 | 7.887584 | 112.61 | -0.9714033 |
| 0 | 36 | 53 | 2 | 33 | 0 | 3 | 4 | 0 | 0 | 0 | 0 | 0 | 1 | 0 | 1 | 1 | 0 | 0.0258799 | 0.0258799 | 0.2366164 | 8.125927 | 98.532 | -0.6550113 |
| 0 | 44 | 1 | 4 | 30 | 0 | 3 | 4 | 0 | 0 | 0 | 0 | 0 | 0 | 0 | 0 | 1 | 0 | 0.0080678 | 0.0080678 | 0.0968132 | 9.425049 | 109.4 | 11.53765 |
| 1 | 10 | 8 | 8 | 23 | 1 | 1 | 4 | 0 | 1 | 0 | 1 | 0 | 1 | 0 | 0 | 1 | 1 | 0.0099835 | 0.0149753 | 0.1198023 | 9.905136 | 106.164 | 1.411475 |
| 1 | 74 | 23 | 6 | 19 | 1 | 1 | 4 | 0 | 1 | 0 | 1 | 1 | 1 | 0 | 1 | 1 | 1 | 0.0243962 | 0.0243962 | 0.2439619 | 8.318499 | 101.425 | 8.693041 |
| 0 | 40 | 28 | 3 | 22 | 1 | 2 | 4 | 0 | 0 | 0 | 0 | 0 | 0 | 0 | 0 | 0 | 0 | 0.02531 | 0.02531 | 0.1518603 | 8.281724 | 112.992 | 0.3146296 |
| 1 | 24 | 40 | 1 | 17 | 1 | 1 | 4 | 0 | 1 | 0 | 1 | 0 | 1 | 1 | 0 | 0 | 0 | 0.0303834 | 0.0331455 | 0.3646006 | 8.012018 | 109 | -1.117464 |
| 1 | 42 | 18 | 3 | 39 | 0 | 4 | 4 | 0 | 0 | 0 | 0 | 1 | 1 | 0 | 0 | 1 | 1 | 0.022298 | 0.0243427 | 0.1217137 | 8.320691 | 106.225 | 0.3884559 |
| 1 | 38 | 34 | 3 | 29 | 1 | 3 | 4 | 0 | 1 | 0 | 1 | 1 | 1 | 0 | 1 | 1 | 1 | 0.015569 | 0.015569 | 0.1556905 | 8.76764 | 108.269 | 1.233363 |
| 1 | 15 | 84 | 5 | 34 | 1 | 3 | 4 | 0 | 0 | 0 | 0 | 0 | 1 | 0 | 1 | 1 | 1 | 0.0174201 | 0.0174201 | 0.0958105 | 9.348449 | 108.064 | 2.942514 |
| 0 | 58 | 97 | 5 | 24 | 1 | 1 | 4 | 0 | 1 | 0 | 1 | 0 | 1 | 0 | 0 | 0 | 0 | 0.0231965 | 0.0231965 | 0.1855718 | 8.368925 | 102.9 | 6.745548 |
| 1 | 85 | 42 | 7 | 37 | 1 | 3 | 4 | 0 | 0 | 0 | 0 | 0 | 1 | 0 | 0 | 1 | 1 | 0.0202476 | 0.0442087 | 0.1768347 | 8.417151 | 106.293 | 2.940821 |
| 1 | 18 | 77 | 1 | 35 | 0 | 6 | 4 | 0 | 0 | 0 | 0 | 0 | 0 | 0 | 1 | 1 | 1 | 0.0261506 | 0.0261506 | 0.1569038 | 8.249052 | 106.479 | 3.396029 |
| 0 | 23 | 71 | 1 | 21 | 1 | 1 | 4 | 0 | 1 | 0 | 1 | 0 | 1 | 0 | 1 | 1 | 0 | 0.0355556 | 0.0177778 | 0.16 | 8.634976 | 106.954 | 0.4955561 |
| 0 | 10 | 3 | 8 | 26 | 1 | 4 | 4 | 1 | 0 | 0 | 0 | 1 | 0 | 0 | 0 | 1 | 0 | 0 | 0 | 0.3475843 | 8.657651 | 100.978 | 4.175152 |
| 0 | 55 | 67 | 5 | 23 | 0 | 2 | 4 | 0 | 0 | 0 | 0 | 0 | 0 | 0 | 0 | 1 | 0 | 0.0300481 | 0.0300481 | 0.2403846 | 8.110126 | 108.391 | 0.2165276 |
| 1 | 18 | 11 | 1 | 28 | 1 | 1 | 1 | 0 | 1 | 0 | 1 | 1 | 1 | 0 | 1 | 1 | 1 | 0.0261506 | 0.0261506 | 0.1569038 | 8.249052 | 106.479 | 3.396029 |
| 0 | 51 | 48 | 4 | 20 | 0 | 1 | 4 | 0 | 1 | 0 | 1 | 1 | 0 | 0 | 1 | 1 | 0 | 0.0140371 | 0.0140371 | 0.1965188 | 8.178078 | 107.939 | -0.5497003 |
| 0 | 40 | 16 | 3 | 33 | 0 | 4 | 4 | 0 | 0 | 0 | 0 | 0 | 0 | 0 | 0 | 0 | 0 | 0.02531 | 0.02531 | 0.1518603 | 8.281724 | 112.992 | 0.3146296 |

| year | Health center | sr | district | Age | Education | Parity | Job | smoking or drug abuse | history of complication in pregnancy | history medical disease in Pregnancy | History of medical or obstetric high risk conditions | complication in this pregnancy | Women’s awareness of the benefits of natural childbirth | Women’s preference for caesarean section | Cesarean section | Intervention (increasein thedensity of midwives) | Interaction between intervention and time | Density of family physicians | Density of Midwives | Density of rural community health (Bhevarz) workers | Logarithm of the rural population | Sex ratio | Socio-economic status |
| --- | --- | --- | --- | --- | --- | --- | --- | --- | --- | --- | --- | --- | --- | --- | --- | --- | --- | --- | --- | --- | --- | --- | --- |
| 1 | 5 | 16 | 7 | 27 | 1 | 1 | 4 | 1 | 1 | 0 | 1 | 0 | 1 | 0 | 1 | 1 | 1 | 0.0176046 | 0.0117364 | 0.0528138 | 9.743378 | 101.549 | 10.75591 |
| 0 | 57 | 102 | 5 | 17 | 1 | 1 | 4 | 0 | 1 | 0 | 1 | 1 | 0 | 0 | 0 | 0 | 0 | 0.0209161 | 0.0209161 | 0.1254968 | 8.472405 | 108.232 | 5.323828 |
| 0 | 38 | 31 | 3 | 33 | 1 | 3 | 4 | 0 | 0 | 0 | 0 | 0 | 1 | 0 | 0 | 1 | 0 | 0 | 0 | 0.1715533 | 8.765926 | 110.299 | 0.0721307 |
| 1 | 17 | 4 | 1 | 21 | 1 | 1 | 4 | 0 | 1 | 0 | 1 | 1 | 1 | 1 | 1 | 1 | 1 | 0.0521921 | 0.026096 | 0.3131524 | 8.251143 | 104.92 | 5.032202 |
| 1 | 42 | 6 | 3 | 24 | 1 | 1 | 4 | 0 | 1 | 0 | 1 | 0 | 1 | 0 | 0 | 1 | 1 | 0.022298 | 0.0243427 | 0.1217137 | 8.320691 | 106.225 | 0.3884559 |
| 0 | 1 | 13 | 2 | 26 | 0 | 2 | 4 | 0 | 1 | 0 | 1 | 0 | 1 | 0 | 0 | 1 | 0 | 0.0123686 | 0.0123686 | 0.148423 | 8.997766 | 102.479 | 9.035476 |
| 0 | 5 | 19 | 7 | 20 | 1 | 1 | 4 | 0 | 1 | 0 | 1 | 0 | 0 | 0 | 0 | 1 | 0 | 0.0127559 | 0.006378 | 0.0574016 | 9.660077 | 104.553 | 10.75591 |
| 1 | 66 | 40 | 8 | 24 | 1 | 1 | 4 | 0 | 1 | 0 | 1 | 0 | 1 | 0 | 0 | 1 | 1 | 0.0225428 | 0.0225428 | 0.1803426 | 8.397509 | 103.114 | -0.4675424 |
| 1 | 45 | 71 | 4 | 41 | 0 | 4 | 4 | 0 | 0 | 0 | 0 | 0 | 1 | 0 | 0 | 1 | 1 | 0.0294774 | 0.0446628 | 0.3573024 | 7.713785 | 107.123 | 14.63733 |
| 1 | 25 | 58 | 1 | 37 | 1 | 3 | 4 | 1 | 0 | 0 | 0 | 0 | 1 | 0 | 0 | 1 | 1 | 0.022604 | 0.022604 | 0.2938517 | 8.394799 | 105.195 | 3.012378 |
| 1 | 20 | 67 | 1 | 34 | 1 | 2 | 1 | 0 | 0 | 0 | 0 | 0 | 1 | 0 | 0 | 0 | 0 | 0.020141 | 0.020141 | 0.2618328 | 8.510169 | 103.234 | -0.099311 |
| 0 | 11 | 53 | 8 | 36 | 0 | 6 | 1 | 0 | 1 | 0 | 1 | 1 | 0 | 0 | 0 | 1 | 0 | 0 | 0 | 0.0964413 | 9.246576 | 107.214 | 1.138871 |
| 0 | 3 | 43 | 6 | 25 | 1 | 1 | 4 | 0 | 1 | 1 | 2 | 1 | 1 | 0 | 0 | 1 | 0 | 0.0105731 | 0.0105731 | 0.2114612 | 9.154616 | 141.399 | -0.8191268 |
| 1 | 14 | 53 | 5 | 34 | 1 | 2 | 4 | 0 | 1 | 0 | 1 | 0 | 0 | 0 | 0 | 1 | 1 | 0.0403986 | 0.0269324 | 0.1481282 | 8.912743 | 106.45 | 6.199605 |
| 1 | 45 | 85 | 4 | 20 | 1 | 1 | 4 | 1 | 1 | 0 | 1 | 0 | 0 | 0 | 0 | 1 | 1 | 0.0294774 | 0.0446628 | 0.3573024 | 7.713785 | 107.123 | 14.63733 |
| 1 | 19 | 90 | 1 | 38 | 0 | 6 | 4 | 0 | 0 | 0 | 0 | 0 | 1 | 0 | 1 | 0 | 0 | 0.0390422 | 0.0520562 | 0.2602811 | 7.560601 | 105.674 | 0.8474746 |
| 1 | 32 | 36 | 2 | 35 | 0 | 2 | 1 | 0 | 0 | 0 | 0 | 1 | 1 | 0 | 0 | 1 | 1 | 0.0364034 | 0.0364034 | 0.2548234 | 7.918265 | 97.9107 | 0.1336359 |
| 0 | 3 | 73 | 6 | 21 | 1 | 1 | 4 | 0 | 1 | 1 | 2 | 0 | 1 | 0 | 0 | 1 | 0 | 0.0105731 | 0.0105731 | 0.2114612 | 9.154616 | 141.399 | -0.8191268 |
| 0 | 52 | 29 | 4 | 36 | 0 | 5 | 4 | 0 | 0 | 0 | 0 | 0 | 1 | 0 | 1 | 0 | 0 | 0.0267502 | 0.0133751 | 0.1681443 | 8.785998 | 109.076 | 5.935247 |
| 0 | 13 | 46 | 5 | 16 | 1 | 1 | 2 | 0 | 1 | 0 | 1 | 1 | 1 | 0 | 0 | 1 | 0 | 0.0125881 | 0.0125881 | 0.0629406 | 8.980172 | 107.849 | 4.278709 |
| 0 | 82 | 9 | 7 | 21 | 1 | 1 | 4 | 0 | 1 | 0 | 1 | 0 | 0 | 0 | 0 | 1 | 0 | 0.0260611 | 0.0130305 | 0.0893522 | 8.812099 | 107.9 | 6.972907 |
| 1 | 47 | 65 | 4 | 27 | 1 | 2 | 4 | 0 | 0 | 0 | 0 | 1 | 1 | 0 | 0 | 0 | 0 | 0.0192752 | 0.0192752 | 0.1349268 | 8.554104 | 109.362 | 6.731958 |

| year | Health center | sr | district | Age | Education | Parity | Job | smoking or drug abuse | history of complication in pregnancy | history medical disease in Pregnancy | History of medical or obstetric high risk conditions | complication in this pregnancy | Women’s awareness of the benefits of natural childbirth | Women’s preference for caesarean section | Cesarean section | Intervention (increasein thedensity of midwives) | Interaction between intervention and time | Density of family physicians | Density of Midwives | Density of rural community health (Bhevarz) workers | Logarithm of the rural population | Sex ratio | Socio-economic status |
| --- | --- | --- | --- | --- | --- | --- | --- | --- | --- | --- | --- | --- | --- | --- | --- | --- | --- | --- | --- | --- | --- | --- | --- |
| 0 | 62 | 64 | 8 | 36 | 1 | 3 | 4 | 1 | 0 | 0 | 0 | 0 | 1 | 0 | 0 | 1 | 0 | 0.010862 | 0.010862 | 0.139034 | 8.657651 | 100.978 | -0.6016933 |
| 0 | 67 | 65 | 8 | 22 | 1 | 1 | 4 | 1 | 1 | 0 | 1 | 1 | 1 | 0 | 1 | 0 | 0 | 0.012238 | 0.0122381 | 0.137067 | 8.538367 | 106.427 | -0.6212724 |
| 0 | 5 | 17 | 7 | 25 | 1 | 2 | 4 | 1 | 0 | 0 | 0 | 1 | 0 | 0 | 1 | 1 | 0 | 0.012756 | 0.006378 | 0.057402 | 9.660077 | 104.553 | 10.75591 |
| 0 | 14 | 80 | 5 | 20 | 1 | 1 | 4 | 0 | 1 | 0 | 1 | 0 | 1 | 0 | 0 | 1 | 0 | 0.017802 | 0.0089008 | 0.142412 | 9.326789 | 119.992 | 6.199605 |
| 1 | 47 | 61 | 4 | 21 | 1 | 1 | 4 | 0 | 1 | 0 | 1 | 0 | 0 | 0 | 0 | 0 | 0 | 0.019275 | 0.0192752 | 0.134927 | 8.554104 | 109.362 | 6.731958 |
| 0 | 18 | 80 | 1 | 22 | 1 | 1 | 4 | 0 | 1 | 0 | 1 | 1 | 1 | 0 | 1 | 1 | 0 | 0 | 0 | 0.094038 | 8.578665 | 102.707 | 2.15365 |
| 1 | 57 | 102 | 5 | 17 | 1 | 1 | 4 | 0 | 1 | 0 | 1 | 0 | 1 | 0 | 0 | 0 | 0 | 0.018514 | 0.0223065 | 0.111533 | 8.408048 | 105.831 | 5.323828 |
| 0 | 38 | 41 | 3 | 29 | 0 | 2 | 1 | 0 | 0 | 0 | 0 | 0 | 0 | 0 | 0 | 1 | 0 | 0 | 0 | 0.171553 | 8.765926 | 110.299 | 0.0721307 |
| 1 | 46 | 41 | 4 | 29 | 1 | 1 | 4 | 1 | 1 | 0 | 1 | 0 | 0 | 0 | 0 | 1 | 1 | 0.046993 | 0.0469925 | 0.18797 | 7.662938 | 105.803 | -0.5406668 |
| 1 | 75 | 47 | 6 | 28 | 1 | 2 | 4 | 0 | 0 | 0 | 0 | 0 | 0 | 0 | 0 | 1 | 1 | 0.025144 | 0.0274499 | 0.219599 | 8.200562 | 104.319 | 0.7997856 |
| 1 | 15 | 18 | 5 | 31 | 0 | 3 | 4 | 0 | 0 | 0 | 0 | 0 | 0 | 0 | 0 | 1 | 1 | 0.01742 | 0.0174201 | 0.095811 | 9.348449 | 108.064 | 2.942514 |
| 0 | 66 | 48 | 8 | 32 | 1 | 3 | 4 | 0 | 1 | 0 | 1 | 1 | 0 | 0 | 1 | 1 | 0 | 0 | 0 | 0.150182 | 8.446985 | 96.1814 | 0.0736284 |
| 0 | 15 | 108 | 5 | 30 | 1 | 1 | 4 | 0 | 1 | 0 | 1 | 0 | 1 | 1 | 1 | 1 | 0 | 0 | 0 | 0.119606 | 9.29367 | 109.503 | 2.01282 |
| 1 | 53 | 50 | 5 | 40 | 1 | 3 | 2 | 0 | 0 | 0 | 0 | 0 | 1 | 1 | 0 | 0 | 0 | 0.027027 | 0.027027 | 0.162162 | 8.216088 | 106.243 | 0.5041221 |
| 0 | 51 | 54 | 4 | 28 | 0 | 3 | 4 | 0 | 1 | 0 | 1 | 1 | 0 | 0 | 0 | 1 | 0 | 0.014037 | 0.0140371 | 0.196519 | 8.178078 | 107.939 | -0.5497003 |
| 0 | 58 | 89 | 5 | 17 | 1 | 1 | 4 | 1 | 1 | 0 | 1 | 0 | 0 | 0 | 1 | 0 | 0 | 0.023197 | 0.0231965 | 0.185572 | 8.368925 | 102.9 | 6.745548 |
| 0 | 7 | 35 | 7 | 31 | 0 | 3 | 4 | 0 | 0 | 0 | 0 | 0 | 0 | 0 | 0 | 1 | 0 | 0.010912 | 0.0095482 | 0.065474 | 9.123038 | 107.143 | 2.840596 |
| 1 | 11 | 75 | 8 | 28 | 1 | 2 | 4 | 0 | 0 | 0 | 0 | 0 | 0 | 0 | 1 | 1 | 1 | 0.019457 | 0.0194571 | 0.107014 | 9.237858 | 104.232 | 0.4590139 |
| 1 | 44 | 5 | 4 | 43 | 1 | 1 | 4 | 0 | 1 | 0 | 1 | 1 | 1 | 0 | 0 | 1 | 1 | 0.011522 | 0.0115221 | 0.138265 | 9.068662 | 107.4 | 11.53765 |
| 0 | 77 | 55 | 6 | 24 | 1 | 2 | 4 | 0 | 1 | 0 | 1 | 0 | 0 | 0 | 0 | 1 | 0 | 0.00732 | 0.0073202 | 0.163973 | 9.052282 | 110.7 | 1.235519 |
| 1 | 57 | 106 | 5 | 41 | 0 | 2 | 4 | 0 | 0 | 0 | 0 | 0 | 0 | 0 | 0 | 0 | 0 | 0.018514 | 0.0223065 | 0.111533 | 8.408048 | 105.831 | 5.323828 |
| 0 | 17 | 86 | 1 | 31 | 0 | 4 | 4 | 0 | 0 | 0 | 0 | 0 | 0 | 0 | 0 | 1 | 0 | 0.018224 | 0.0145794 | 0.116635 | 8.833317 | 105.359 | 1.309201 |

| year | Health center | sr | district | Age | Education | Parity | Job | smoking or drug abuse | history of complication in pregnancy | history medical disease in Pregnancy | History of medical or obstetric high risk conditions | complication in this pregnancy | Women’s awareness of the benefits of natural childbirth | Women’s preference for caesarean section | Cesarean section | Intervention (increasein thedensity of midwives) | Interaction between intervention and time | Density of family physicians | Density of Midwives | Density of rural community health (Bhevarz) workers | Logarithm of the rural population | Sex ratio | Socio-economic status |
| --- | --- | --- | --- | --- | --- | --- | --- | --- | --- | --- | --- | --- | --- | --- | --- | --- | --- | --- | --- | --- | --- | --- | --- |
| 1 | 56 | 7 | 5 | 33 | 0 | 2 | 4 | 0 | 0 | 0 | 0 | 0 | 1 | 0 | 0 | 1 | 1 | 0.03076 | 0.0307598 | 0.184559 | 8.086718 | 110.83 | 5.878881 |
| 1 | 19 | 13 | 1 | 22 | 1 | 2 | 4 | 0 | 0 | 0 | 0 | 1 | 1 | 1 | 0 | 0 | 0 | 0.039042 | 0.0520562 | 0.260281 | 7.560601 | 105.674 | 0.8474746 |
| 1 | 23 | 35 | 1 | 24 | 1 | 2 | 1 | 0 | 0 | 0 | 0 | 0 | 0 | 0 | 0 | 1 | 1 | 0.022568 | 0.0225683 | 0.270819 | 8.39638 | 103.631 | 3.133176 |
| 0 | 8 | 38 | 4 | 29 | 1 | 2 | 4 | 0 | 0 | 0 | 0 | 0 | 1 | 0 | 0 | 1 | 0 | 0.01042 | 0.0104199 | 0.156299 | 9.169206 | 109.267 | 2.423543 |
| 0 | 30 | 73 | 2 | 36 | 0 | 3 | 4 | 1 | 0 | 0 | 0 | 0 | 1 | 0 | 0 | 1 | 0 | 0.020124 | 0.0201242 | 0.160994 | 8.377471 | 101.296 | 9.05479 |
| 1 | 10 | 69 | 8 | 31 | 1 | 3 | 1 | 0 | 0 | 0 | 0 | 0 | 0 | 0 | 0 | 1 | 1 | 0.009984 | 0.0149753 | 0.119802 | 9.905136 | 106.164 | 1.411475 |
| 0 | 15 | 13 | 5 | 32 | 1 | 3 | 4 | 1 | 1 | 0 | 1 | 1 | 0 | 0 | 0 | 1 | 0 | 0 | 0 | 0.119606 | 9.29367 | 109.503 | 2.01282 |
| 0 | 17 | 84 | 1 | 20 | 1 | 1 | 4 | 0 | 1 | 0 | 1 | 0 | 1 | 0 | 0 | 1 | 0 | 0.018224 | 0.0145794 | 0.116635 | 8.833317 | 105.359 | 1.309201 |
| 1 | 51 | 52 | 4 | 34 | 1 | 2 | 4 | 0 | 1 | 0 | 1 | 1 | 1 | 0 | 0 | 1 | 1 | 0.02951 | 0.0322165 | 0.225516 | 8.040447 | 110.583 | 0.1859285 |
| 0 | 15 | 109 | 5 | 22 | 1 | 2 | 4 | 0 | 0 | 0 | 0 | 0 | 0 | 0 | 0 | 1 | 0 | 0 | 0 | 0.119606 | 9.29367 | 109.503 | 2.01282 |
| 0 | 58 | 95 | 5 | 37 | 0 | 5 | 4 | 0 | 0 | 0 | 0 | 0 | 0 | 0 | 0 | 0 | 0 | 0.023197 | 0.0231965 | 0.185572 | 8.368925 | 102.9 | 6.745548 |
| 0 | 18 | 12 | 1 | 27 | 0 | 2 | 4 | 1 | 0 | 0 | 0 | 1 | 0 | 0 | 1 | 1 | 0 | 0 | 0 | 0.094038 | 8.578665 | 102.707 | 2.15365 |
| 0 | 25 | 53 | 1 | 26 | 1 | 2 | 4 | 0 | 1 | 0 | 1 | 0 | 1 | 0 | 0 | 1 | 0 | 0.022254 | 0.0178031 | 0.195834 | 8.633553 | 106.356 | 2.158408 |
| 0 | 47 | 58 | 4 | 34 | 0 | 6 | 4 | 0 | 1 | 0 | 1 | 1 | 0 | 0 | 0 | 0 | 0 | 0.019489 | 0.0194894 | 0.077958 | 8.543056 | 108.408 | 2.371052 |
| 1 | 30 | 72 | 2 | 33 | 1 | 3 | 4 | 0 | 0 | 0 | 0 | 0 | 1 | 0 | 0 | 1 | 1 | 0.025507 | 0.0307314 | 0.153657 | 8.087641 | 101.486 | 11.04882 |
| 1 | 10 | 70 | 8 | 29 | 1 | 2 | 1 | 0 | 0 | 0 | 0 | 0 | 1 | 0 | 0 | 1 | 1 | 0.009984 | 0.0149753 | 0.119802 | 9.905136 | 106.164 | 1.411475 |
| 0 | 58 | 94 | 5 | 19 | 1 | 1 | 4 | 1 | 1 | 0 | 1 | 1 | 0 | 0 | 0 | 0 | 0 | 0.023197 | 0.0231965 | 0.185572 | 8.368925 | 102.9 | 6.745548 |
| 0 | 14 | 34 | 5 | 25 | 0 | 2 | 1 | 0 | 0 | 0 | 0 | 1 | 1 | 0 | 0 | 1 | 0 | 0.017802 | 0.0089008 | 0.142412 | 9.326789 | 119.992 | 6.199605 |
| 1 | 52 | 16 | 4 | 31 | 1 | 1 | 4 | 0 | 1 | 0 | 1 | 1 | 1 | 1 | 0 | 0 | 0 | 0.015482 | 0.0154823 | 0.185787 | 8.77323 | 106.16 | 2.028297 |
| 0 | 15 | 114 | 5 | 14 | 0 | 1 | 4 | 0 | 1 | 0 | 1 | 1 | 1 | 0 | 0 | 1 | 0 | 0 | 0 | 0.119606 | 9.29367 | 109.503 | 2.01282 |
| 1 | 20 | 22 | 1 | 23 | 1 | 2 | 4 | 0 | 0 | 0 | 0 | 0 | 0 | 0 | 0 | 0 | 0 | 0.020141 | 0.020141 | 0.261833 | 8.510169 | 103.234 | -0.099311 |
| 0 | 11 | 81 | 8 | 26 | 0 | 3 | 4 | 0 | 0 | 0 | 0 | 0 | 1 | 0 | 0 | 1 | 0 | 0 | 0 | 0.096441 | 9.246576 | 107.214 | 1.138871 |

| year | Health center | sr | district | Age | Education | Parity | Job | smoking or drug abuse | history of complication in pregnancy | history medical disease in Pregnancy | History of medical or obstetric high risk conditions | complication in this pregnancy | Women’s awareness of the benefits of natural childbirth | Women’s preference for caesarean section | Cesarean section | Intervention (increasein thedensity of midwives) | Interaction between intervention and time | Density of family physicians | Density of Midwives | Density of rural community health (Bhevarz) workers | Logarithm of the rural population | Sex ratio | Socio-economic status |
| --- | --- | --- | --- | --- | --- | --- | --- | --- | --- | --- | --- | --- | --- | --- | --- | --- | --- | --- | --- | --- | --- | --- | --- |
| 0 | 40 | 25 | 3 | 38 | 0 | 8 | 4 | 0 | 0 | 0 | 0 | 0 | 0 | 0 | 0 | 0 | 0 | 0.02531 | 0.02531 | 0.15186 | 8.281724 | 112.992 | 0.3146296 |
| 0 | 5 | 21 | 7 | 18 | 1 | 1 | 4 | 0 | 1 | 0 | 1 | 0 | 0 | 0 | 0 | 1 | 0 | 0.012756 | 0.006378 | 0.057402 | 9.660077 | 104.553 | 10.75591 |
| 1 | 5 | 14 | 7 | 34 | 0 | 3 | 4 | 0 | 0 | 1 | 1 | 0 | 1 | 0 | 0 | 1 | 1 | 0.017605 | 0.0117364 | 0.052814 | 9.743378 | 101.549 | 10.75591 |
| 0 | 39 | 37 | 3 | 26 | 1 | 4 | 4 | 0 | 0 | 0 | 0 | 1 | 0 | 0 | 0 | 1 | 0 | 0.023703 | 0.0165924 | 0.142221 | 9.263597 | 107 | 2.934642 |
| 0 | 83 | 2 | 7 | 30 | 0 | 1 | 4 | 0 | 1 | 0 | 1 | 1 | 1 | 0 | 0 | 0 | 0 | 0.016647 | 0.0166472 | 0.133178 | 8.700681 | 106.001 | 2.64662 |
| 0 | 14 | 107 | 5 | 30 | 0 | 3 | 4 | 1 | 0 | 0 | 0 | 1 | 0 | 0 | 0 | 1 | 0 | 0.017802 | 0.0089008 | 0.142412 | 9.326789 | 119.992 | 6.199605 |
| 0 | 23 | 70 | 1 | 23 | 1 | 1 | 4 | 0 | 1 | 0 | 1 | 0 | 0 | 0 | 0 | 1 | 0 | 0.035556 | 0.0177778 | 0.16 | 8.634976 | 106.954 | 0.4955561 |
| 1 | 14 | 33 | 5 | 27 | 1 | 1 | 4 | 0 | 1 | 0 | 1 | 1 | 1 | 1 | 1 | 1 | 1 | 0.040399 | 0.0269324 | 0.148128 | 8.912743 | 106.45 | 6.199605 |
| 0 | 14 | 33 | 5 | 20 | 1 | 1 | 4 | 0 | 1 | 0 | 1 | 1 | 0 | 0 | 1 | 1 | 0 | 0.017802 | 0.0089008 | 0.142412 | 9.326789 | 119.992 | 6.199605 |
| 0 | 72 | 31 | 6 | 27 | 0 | 2 | 4 | 0 | 0 | 0 | 0 | 0 | 0 | 0 | 0 | 1 | 0 | 0.012609 | 0.0105077 | 0.201749 | 8.69081 | 104.258 | 1.426343 |
| 1 | 54 | 43 | 5 | 24 | 1 | 1 | 4 | 0 | 1 | 0 | 1 | 0 | 1 | 0 | 1 | 1 | 1 | 0.013163 | 0.01755 | 0.15795 | 8.64787 | 108.032 | -0.2427101 |
| 0 | 29 | 4 | 2 | 30 | 1 | 4 | 1 | 1 | 0 | 0 | 0 | 1 | 0 | 0 | 0 | 1 | 0 | 0.017372 | 0.0173715 | 0.138972 | 8.524566 | 104.01 | 5.093501 |
| 1 | 30 | 69 | 2 | 25 | 1 | 2 | 1 | 0 | 0 | 0 | 0 | 1 | 1 | 0 | 0 | 1 | 1 | 0.025507 | 0.0307314 | 0.153657 | 8.087641 | 101.486 | 11.04882 |
| 1 | 14 | 107 | 5 | 38 | 1 | 5 | 4 | 0 | 0 | 0 | 0 | 1 | 0 | 0 | 0 | 1 | 1 | 0.040399 | 0.0269324 | 0.148128 | 8.912743 | 106.45 | 6.199605 |
| 0 | 84 | 26 | 7 | 38 | 0 | 7 | 4 | 0 | 0 | 0 | 0 | 1 | 1 | 0 | 1 | 1 | 0 | 0.022454 | 0.017963 | 0.251482 | 8.624612 | 110.07 | 4.268024 |
| 1 | 3 | 42 | 6 | 32 | 1 | 2 | 4 | 0 | 0 | 0 | 0 | 0 | 1 | 1 | 0 | 1 | 1 | 0.049925 | 0.0499251 | 0.524214 | 8.295548 | 104.806 | 1.110284 |
| 0 | 74 | 14 | 6 | 24 | 0 | 1 | 4 | 0 | 1 | 0 | 1 | 0 | 0 | 0 | 0 | 1 | 0 | 0.027489 | 0.014994 | 0.219912 | 8.517593 | 101.45 | 1.682292 |
| 1 | 34 | 48 | 2 | 24 | 1 | 2 | 4 | 0 | 0 | 0 | 0 | 0 | 1 | 1 | 0 | 0 | 0 | 0.042626 | 0.0426257 | 0.255755 | 7.760467 | 104.534 | 1.028819 |
| 1 | 39 | 37 | 3 | 21 | 1 | 2 | 4 | 0 | 0 | 0 | 0 | 0 | 0 | 0 | 1 | 1 | 1 | 0.01992 | 0.0398406 | 0.418327 | 8.521185 | 107.7 | 2.925639 |
| 0 | 38 | 47 | 3 | 32 | 0 | 3 | 4 | 0 | 1 | 1 | 2 | 0 | 0 | 0 | 0 | 1 | 0 | 0 | 0 | 0.171553 | 8.765926 | 110.299 | 0.0721307 |
| 1 | 17 | 82 | 1 | 24 | 1 | 3 | 4 | 0 | 0 | 0 | 0 | 1 | 1 | 0 | 1 | 1 | 1 | 0.052192 | 0.026096 | 0.313152 | 8.251143 | 104.92 | 5.032202 |
| 0 | 81 | 30 | 7 | 32 | 1 | 1 | 4 | 0 | 1 | 0 | 1 | 0 | 0 | 0 | 0 | 1 | 0 | 0.009667 | 0.0193349 | 0.135344 | 8.551015 | 106.385 | 9.337543 |

| year | Health center | sr | district | Age | Education | Parity | Job | smoking or drug abuse | history of complication in pregnancy | history medical disease in Pregnancy | History of medical or obstetric high risk conditions | complication in this pregnancy | Women’s awareness of the benefits of natural childbirth | Women’s preference for caesarean section | Cesarean section | Intervention (increasein thedensity of midwives) | Interaction between intervention and time | Density of family physicians | Density of Midwives | Density of rural community health (Bhevarz) workers | Logarithm of the rural population | Sex ratio | Socio-economic status |
| --- | --- | --- | --- | --- | --- | --- | --- | --- | --- | --- | --- | --- | --- | --- | --- | --- | --- | --- | --- | --- | --- | --- | --- |
| 0 | 39 | 40 | 3 | 19 | 1 | 1 | 4 | 0 | 1 | 0 | 1 | 0 | 1 | 1 | 0 | 1 | 0 | 0.023703 | 0.0165924 | 0.142221 | 9.263597 | 107 | 2.934642 |
| 1 | 10 | 4 | 8 | 36 | 0 | 3 | 4 | 0 | 0 | 0 | 0 | 1 | 0 | 0 | 0 | 1 | 1 | 0.009984 | 0.0149753 | 0.119802 | 9.905136 | 106.164 | 1.411475 |
| 1 | 12 | 15 | 3 | 29 | 1 | 1 | 1 | 0 | 1 | 0 | 1 | 0 | 0 | 0 | 1 | 1 | 1 | 0.025468 | 0.025468 | 0.165542 | 8.968651 | 109.525 | -0.2710156 |
| 0 | 21 | 25 | 1 | 20 | 1 | 1 | 4 | 0 | 1 | 0 | 1 | 1 | 1 | 1 | 1 | 0 | 0 | 0.02622 | 0.0190694 | 0.095347 | 8.56484 | 102.315 | -1.117464 |
| 0 | 77 | 56 | 6 | 28 | 1 | 2 | 4 | 1 | 0 | 0 | 0 | 0 | 0 | 0 | 0 | 1 | 0 | 0.00732 | 0.0073202 | 0.163973 | 9.052282 | 110.7 | 1.235519 |
| 1 | 18 | 80 | 1 | 40 | 0 | 3 | 4 | 0 | 0 | 0 | 0 | 0 | 1 | 1 | 1 | 1 | 1 | 0.026151 | 0.0261506 | 0.156904 | 8.249052 | 106.479 | 3.396029 |
| 0 | 66 | 43 | 8 | 28 | 0 | 1 | 4 | 0 | 1 | 0 | 1 | 1 | 1 | 0 | 0 | 1 | 0 | 0 | 0 | 0.150182 | 8.446985 | 96.1814 | 0.0736284 |
| 0 | 14 | 71 | 5 | 26 | 1 | 2 | 4 | 1 | 0 | 0 | 0 | 0 | 0 | 0 | 0 | 1 | 0 | 0.017802 | 0.0089008 | 0.142412 | 9.326789 | 119.992 | 6.199605 |
| 0 | 36 | 57 | 2 | 25 | 1 | 1 | 2 | 0 | 1 | 0 | 1 | 1 | 0 | 0 | 0 | 1 | 0 | 0.02588 | 0.0258799 | 0.236616 | 8.125927 | 98.532 | -0.6550113 |
| 0 | 18 | 11 | 1 | 22 | 0 | 2 | 4 | 0 | 0 | 0 | 0 | 1 | 0 | 0 | 0 | 1 | 0 | 0 | 0 | 0.094038 | 8.578665 | 102.707 | 2.15365 |
| 0 | 74 | 24 | 6 | 18 | 1 | 1 | 4 | 0 | 1 | 0 | 1 | 1 | 1 | 1 | 1 | 1 | 0 | 0.027489 | 0.014994 | 0.219912 | 8.517593 | 101.45 | 1.682292 |
| 1 | 45 | 73 | 4 | 32 | 0 | 5 | 4 | 0 | 0 | 0 | 0 | 0 | 1 | 0 | 0 | 1 | 1 | 0.029477 | 0.0446628 | 0.357302 | 7.713785 | 107.123 | 14.63733 |
| 1 | 11 | 50 | 8 | 28 | 1 | 1 | 4 | 0 | 1 | 0 | 1 | 1 | 1 | 0 | 0 | 1 | 1 | 0.019457 | 0.0194571 | 0.107014 | 9.237858 | 104.232 | 0.4590139 |
| 0 | 34 | 42 | 2 | 31 | 0 | 2 | 4 | 0 | 0 | 0 | 0 | 0 | 0 | 0 | 0 | 0 | 0 | 0.032944 | 0.0329443 | 0.150602 | 7.884576 | 101.977 | 2.049861 |
| 1 | 11 | 54 | 8 | 20 | 1 | 1 | 4 | 0 | 1 | 0 | 1 | 0 | 0 | 0 | 0 | 1 | 1 | 0.019457 | 0.0194571 | 0.107014 | 9.237858 | 104.232 | 0.4590139 |
| 1 | 3 | 69 | 6 | 26 | 1 | 1 | 4 | 0 | 1 | 0 | 1 | 1 | 1 | 0 | 0 | 1 | 1 | 0.049925 | 0.0499251 | 0.524214 | 8.295548 | 104.806 | 1.110284 |
| 0 | 8 | 12 | 4 | 34 | 0 | 6 | 4 | 1 | 1 | 1 | 2 | 1 | 1 | 1 | 1 | 1 | 0 | 0.01042 | 0.0104199 | 0.156299 | 9.169206 | 109.267 | 2.423543 |
| 1 | 1 | 17 | 2 | 30 | 1 | 1 | 3 | 0 | 1 | 0 | 1 | 1 | 0 | 0 | 0 | 1 | 1 | 0.061843 | 0.0412286 | 0.247372 | 8.48694 | 99.5475 | 10.18861 |
| 1 | 11 | 77 | 8 | 26 | 1 | 3 | 4 | 0 | 0 | 0 | 0 | 0 | 0 | 0 | 0 | 1 | 1 | 0.019457 | 0.0194571 | 0.107014 | 9.237858 | 104.232 | 0.4590139 |
| 0 | 36 | 95 | 2 | 28 | 0 | 2 | 4 | 0 | 0 | 1 | 1 | 1 | 0 | 0 | 1 | 1 | 0 | 0.02588 | 0.0258799 | 0.236616 | 8.125927 | 98.532 | -0.6550113 |
| 0 | 8 | 7 | 4 | 36 | 0 | 5 | 4 | 0 | 1 | 0 | 1 | 0 | 0 | 0 | 0 | 1 | 0 | 0.01042 | 0.0104199 | 0.156299 | 9.169206 | 109.267 | 2.423543 |
| 1 | 5 | 15 | 7 | 32 | 1 | 3 | 4 | 0 | 0 | 0 | 0 | 0 | 1 | 1 | 0 | 1 | 1 | 0.017605 | 0.0117364 | 0.052814 | 9.743378 | 101.549 | 10.75591 |

| year | Health center | sr | district | Age | Education | Parity | Job | smoking or drug abuse | history of complication in pregnancy | history medical disease in Pregnancy | History of medical or obstetric high risk conditions | complication in this pregnancy | Women’s awareness of the benefits of natural childbirth | Women’s preference for caesarean section | Cesarean section | Intervention (increasein thedensity of midwives) | Interaction between intervention and time | Density of family physicians | Density of Midwives | Density of rural community health (Bhevarz) workers | Logarithm of the rural population | Sex ratio | Socio-economic status |
| --- | --- | --- | --- | --- | --- | --- | --- | --- | --- | --- | --- | --- | --- | --- | --- | --- | --- | --- | --- | --- | --- | --- | --- |
| 1 | 42 | 5 | 3 | 35 | 0 | 4 | 1 | 0 | 0 | 0 | 0 | 0 | 0 | 0 | 0 | 1 | 1 | 0.022298 | 0.0243427 | 0.121714 | 8.320691 | 106.225 | 0.3884559 |
| 1 | 1 | 89 | 2 | 21 | 1 | 1 | 4 | 0 | 1 | 1 | 2 | 1 | 1 | 0 | 0 | 1 | 1 | 0.061843 | 0.0412286 | 0.247372 | 8.48694 | 99.5475 | 10.18861 |
| 0 | 10 | 59 | 8 | 34 | 0 | 4 | 4 | 1 | 0 | 0 | 0 | 0 | 0 | 0 | 1 | 1 | 0 | 0 | 0 | 0.347584 | 8.657651 | 100.978 | 4.175152 |
| 1 | 21 | 30 | 1 | 24 | 1 | 1 | 4 | 0 | 1 | 1 | 2 | 0 | 1 | 0 | 1 | 0 | 0 | 0.020194 | 0.0201939 | 0.201939 | 8.507546 | 104.375 | -1.034636 |
| 0 | 4 | 3 | 6 | 32 | 1 | 3 | 4 | 0 | 0 | 1 | 1 | 1 | 1 | 0 | 1 | 1 | 0 | 0.01162 | 0.0116198 | 0.185917 | 9.060215 | 108.479 | 1.503687 |
| 1 | 50 | 92 | 4 | 39 | 1 | 4 | 4 | 0 | 0 | 0 | 0 | 1 | 0 | 0 | 0 | 1 | 1 | 0.02004 | 0.0200401 | 0.140281 | 8.515191 | 102.599 | 1.292794 |
| 0 | 13 | 42 | 5 | 30 | 0 | 4 | 4 | 0 | 1 | 1 | 2 | 1 | 0 | 0 | 1 | 1 | 0 | 0.012588 | 0.0125881 | 0.062941 | 8.980172 | 107.849 | 4.278709 |
| 0 | 34 | 100 | 2 | 32 | 0 | 2 | 4 | 0 | 0 | 0 | 0 | 0 | 1 | 1 | 1 | 0 | 0 | 0.032944 | 0.0329443 | 0.150602 | 7.884576 | 101.977 | 2.049861 |
| 1 | 45 | 67 | 4 | 24 | 1 | 1 | 4 | 0 | 1 | 0 | 1 | 0 | 0 | 0 | 0 | 1 | 1 | 0.029477 | 0.0446628 | 0.357302 | 7.713785 | 107.123 | 14.63733 |
| 0 | 5 | 15 | 7 | 19 | 1 | 1 | 4 | 0 | 1 | 0 | 1 | 0 | 0 | 0 | 0 | 1 | 0 | 0.012756 | 0.006378 | 0.057402 | 9.660077 | 104.553 | 10.75591 |
| 1 | 74 | 14 | 6 | 35 | 1 | 3 | 4 | 0 | 0 | 0 | 0 | 0 | 0 | 0 | 0 | 1 | 1 | 0.024396 | 0.0243962 | 0.243962 | 8.318499 | 101.425 | 8.693041 |
| 0 | 3 | 69 | 6 | 24 | 1 | 2 | 4 | 0 | 0 | 0 | 0 | 0 | 0 | 0 | 0 | 1 | 0 | 0.010573 | 0.0105731 | 0.211461 | 9.154616 | 141.399 | -0.8191268 |
| 0 | 45 | 86 | 4 | 37 | 0 | 2 | 4 | 1 | 0 | 0 | 0 | 0 | 0 | 0 | 1 | 1 | 0 | 0.022355 | 0.0223547 | 0.23845 | 8.118207 | 107.997 | 0.1683084 |
| 1 | 20 | 65 | 1 | 33 | 1 | 5 | 4 | 0 | 0 | 0 | 0 | 1 | 1 | 0 | 0 | 0 | 0 | 0.020141 | 0.020141 | 0.261833 | 8.510169 | 103.234 | -0.099311 |
| 1 | 63 | 1 | 8 | 23 | 1 | 2 | 4 | 0 | 0 | 1 | 1 | 1 | 0 | 0 | 1 | 1 | 1 | 0.02649 | 0.0264901 | 0.238411 | 8.236156 | 106.51 | -0.5511303 |
| 1 | 13 | 40 | 5 | 26 | 1 | 2 | 3 | 0 | 0 | 0 | 0 | 0 | 1 | 1 | 0 | 1 | 1 | 0.016941 | 0.0256674 | 0.205339 | 8.267706 | 105.485 | 4.942792 |
| 1 | 18 | 9 | 1 | 26 | 1 | 1 | 4 | 0 | 1 | 0 | 1 | 1 | 1 | 0 | 0 | 1 | 1 | 0.026151 | 0.0261506 | 0.156904 | 8.249052 | 106.479 | 3.396029 |
| 1 | 57 | 99 | 5 | 31 | 1 | 2 | 4 | 0 | 0 | 0 | 0 | 0 | 1 | 1 | 1 | 0 | 0 | 0.018514 | 0.0223065 | 0.111533 | 8.408048 | 105.831 | 5.323828 |
| 0 | 20 | 69 | 1 | 21 | 1 | 2 | 4 | 0 | 0 | 0 | 0 | 0 | 0 | 0 | 0 | 0 | 0 | 0.029264 | 0.0180083 | 0.162075 | 8.622093 | 105.819 | -0.5354715 |
| 0 | 84 | 28 | 7 | 29 | 1 | 3 | 4 | 0 | 1 | 0 | 1 | 1 | 0 | 0 | 0 | 1 | 0 | 0.022454 | 0.017963 | 0.251482 | 8.624612 | 110.07 | 4.268024 |
| 1 | 52 | 27 | 4 | 34 | 1 | 2 | 4 | 0 | 0 | 0 | 0 | 1 | 0 | 0 | 1 | 0 | 0 | 0.015482 | 0.0154823 | 0.185787 | 8.77323 | 106.16 | 2.028297 |
| 0 | 29 | 12 | 2 | 29 | 1 | 3 | 4 | 0 | 0 | 0 | 0 | 0 | 0 | 0 | 0 | 1 | 0 | 0.017372 | 0.0173715 | 0.138972 | 8.524566 | 104.01 | 5.093501 |

| year | Health center | sr | district | Age | Education | Parity | Job | smoking or drug abuse | history of complication in pregnancy | history medical disease in Pregnancy | History of medical or obstetric high risk conditions | complication in this pregnancy | Women’s awareness of the benefits of natural childbirth | Women’s preference for caesarean section | Cesarean section | Intervention (increasein thedensity of midwives) | Interaction between intervention and time | Density of family physicians | Density of Midwives | Density of rural community health (Bhevarz) workers | Logarithm of the rural population | Sex ratio | Socio-economic status |
| --- | --- | --- | --- | --- | --- | --- | --- | --- | --- | --- | --- | --- | --- | --- | --- | --- | --- | --- | --- | --- | --- | --- | --- |
| 1 | 37 | 60 | 2 | 29 | 0 | 1 | 4 | 0 | 1 | 0 | 1 | 1 | 1 | 0 | 1 | 1 | 1 | 0.029274 | 0.029274 | 0.263466 | 8.136226 | 104.796 | 0.399502 |
| 0 | 52 | 18 | 4 | 36 | 0 | 4 | 4 | 1 | 0 | 0 | 0 | 0 | 1 | 0 | 0 | 0 | 0 | 0.02675 | 0.0133751 | 0.168144 | 8.785998 | 109.076 | 5.935247 |
| 0 | 34 | 47 | 2 | 27 | 0 | 2 | 4 | 0 | 0 | 0 | 0 | 0 | 1 | 0 | 0 | 0 | 0 | 0.032944 | 0.0329443 | 0.150602 | 7.884576 | 101.977 | 2.049861 |
| 1 | 35 | 52 | 2 | 25 | 1 | 1 | 4 | 0 | 1 | 0 | 1 | 0 | 0 | 0 | 0 | 1 | 1 | 0.013489 | 0.0324254 | 0.259403 | 8.033982 | 98.0732 | 1.267399 |
| 1 | 16 | 4 | 7 | 35 | 1 | 3 | 4 | 0 | 0 | 0 | 0 | 1 | 1 | 0 | 0 | 1 | 1 | 0.018799 | 0.0187988 | 0.225585 | 9.272282 | 106.022 | 3.110565 |
| 1 | 23 | 37 | 1 | 28 | 1 | 2 | 4 | 0 | 0 | 1 | 1 | 1 | 1 | 1 | 0 | 1 | 1 | 0.022568 | 0.0225683 | 0.270819 | 8.39638 | 103.631 | 3.133176 |
| 0 | 45 | 84 | 4 | 20 | 1 | 1 | 4 | 0 | 1 | 0 | 1 | 0 | 0 | 0 | 0 | 1 | 0 | 0.022355 | 0.0223547 | 0.23845 | 8.118207 | 107.997 | 0.1683084 |
| 1 | 72 | 31 | 6 | 29 | 1 | 1 | 4 | 0 | 1 | 0 | 1 | 0 | 0 | 0 | 0 | 1 | 1 | 0.019992 | 0.019992 | 0.19992 | 8.517593 | 104.916 | 7.820109 |
| 1 | 17 | 87 | 1 | 31 | 1 | 3 | 4 | 0 | 0 | 0 | 0 | 1 | 0 | 0 | 1 | 1 | 1 | 0.052192 | 0.026096 | 0.313152 | 8.251143 | 104.92 | 5.032202 |
| 0 | 8 | 11 | 4 | 27 | 1 | 1 | 4 | 0 | 1 | 0 | 1 | 0 | 0 | 0 | 0 | 1 | 0 | 0.01042 | 0.0104199 | 0.156299 | 9.169206 | 109.267 | 2.423543 |
| 0 | 1 | 83 | 2 | 31 | 1 | 1 | 4 | 0 | 1 | 1 | 2 | 1 | 1 | 0 | 0 | 1 | 0 | 0.012369 | 0.0123686 | 0.148423 | 8.997766 | 102.479 | 9.035476 |
| 1 | 34 | 43 | 2 | 19 | 1 | 1 | 4 | 0 | 1 | 0 | 1 | 1 | 1 | 0 | 0 | 0 | 0 | 0.042626 | 0.0426257 | 0.255755 | 7.760467 | 104.534 | 1.028819 |
| 1 | 51 | 51 | 4 | 19 | 1 | 1 | 4 | 0 | 1 | 0 | 1 | 1 | 0 | 0 | 0 | 1 | 1 | 0.02951 | 0.0322165 | 0.225516 | 8.040447 | 110.583 | 0.1859285 |
| 1 | 34 | 47 | 2 | 40 | 0 | 3 | 4 | 0 | 0 | 0 | 0 | 0 | 1 | 0 | 1 | 0 | 0 | 0.042626 | 0.0426257 | 0.255755 | 7.760467 | 104.534 | 1.028819 |
| 0 | 15 | 110 | 5 | 27 | 1 | 3 | 4 | 1 | 0 | 0 | 0 | 0 | 0 | 0 | 0 | 1 | 0 | 0 | 0 | 0.119606 | 9.29367 | 109.503 | 2.01282 |
| 1 | 84 | 27 | 7 | 30 | 1 | 1 | 3 | 0 | 1 | 0 | 1 | 1 | 1 | 0 | 1 | 1 | 1 | 0.055793 | 0.0371955 | 0.334759 | 8.589886 | 107.1 | 4.460475 |
| 0 | 10 | 7 | 8 | 22 | 1 | 1 | 4 | 0 | 1 | 0 | 1 | 0 | 1 | 1 | 0 | 1 | 0 | 0 | 0 | 0.347584 | 8.657651 | 100.978 | 4.175152 |
| 1 | 77 | 54 | 6 | 27 | 1 | 3 | 4 | 0 | 0 | 0 | 0 | 0 | 1 | 0 | 0 | 1 | 1 | 0.033367 | 0.0333667 | 0.233567 | 8.698514 | 108.2 | -0.4198327 |
| 0 | 75 | 51 | 6 | 17 | 1 | 1 | 4 | 0 | 1 | 0 | 1 | 1 | 0 | 0 | 1 | 1 | 0 | 0.003692 | 0.0036917 | 0.184584 | 8.127404 | 104.84 | -0.2603674 |
| 1 | 3 | 27 | 6 | 29 | 1 | 2 | 4 | 0 | 0 | 0 | 0 | 0 | 1 | 0 | 1 | 1 | 1 | 0.049925 | 0.0499251 | 0.524214 | 8.295548 | 104.806 | 1.110284 |
| 0 | 57 | 101 | 5 | 15 | 1 | 1 | 4 | 0 | 1 | 0 | 1 | 0 | 0 | 0 | 0 | 0 | 0 | 0.020916 | 0.0209161 | 0.125497 | 8.472405 | 108.232 | 5.323828 |
| 1 | 38 | 45 | 3 | 21 | 1 | 1 | 4 | 0 | 1 | 0 | 1 | 1 | 0 | 0 | 0 | 1 | 1 | 0.015569 | 0.015569 | 0.155691 | 8.76764 | 108.269 | 1.233363 |

| year | Health center | sr | district | Age | Education | Parity | Job | smoking or drug abuse | history of complication in pregnancy | history medical disease in Pregnancy | History of medical or obstetric high risk conditions | complication in this pregnancy | Women’s awareness of the benefits of natural childbirth | Women’s preference for caesarean section | Cesarean section | Intervention (increasein thedensity of midwives) | Interaction between intervention and time | Density of family physicians | Density of Midwives | Density of rural community health (Bhevarz) workers | Logarithm of the rural population | Sex ratio | Socio-economic status |
| --- | --- | --- | --- | --- | --- | --- | --- | --- | --- | --- | --- | --- | --- | --- | --- | --- | --- | --- | --- | --- | --- | --- | --- |
| 1 | 10 | 28 | 8 | 23 | 1 | 1 | 4 | 0 | 1 | 0 | 1 | 1 | 0 | 0 | 0 | 1 | 1 | 0.009984 | 0.0149753 | 0.119802 | 9.905136 | 106.164 | 1.411475 |
| 1 | 66 | 42 | 8 | 32 | 1 | 7 | 4 | 0 | 0 | 0 | 0 | 0 | 0 | 0 | 0 | 1 | 1 | 0.022543 | 0.0225428 | 0.180343 | 8.397509 | 103.114 | -0.4675424 |
| 1 | 66 | 45 | 8 | 23 | 1 | 2 | 4 | 0 | 1 | 0 | 1 | 0 | 1 | 0 | 1 | 1 | 1 | 0.022543 | 0.0225428 | 0.180343 | 8.397509 | 103.114 | -0.4675424 |
| 1 | 2 | 8 | 6 | 26 | 1 | 1 | 4 | 0 | 1 | 0 | 1 | 0 | 0 | 0 | 1 | 1 | 1 | 0.015221 | 0.0304414 | 0.258752 | 8.790269 | 106.539 | 0.4487849 |
| 1 | 82 | 11 | 7 | 18 | 1 | 1 | 4 | 0 | 1 | 1 | 2 | 0 | 0 | 0 | 1 | 1 | 1 | 0.019051 | 0.0190512 | 0.15241 | 8.565793 | 109.7 | 7.266544 |
| 1 | 13 | 38 | 5 | 26 | 1 | 2 | 4 | 0 | 0 | 0 | 0 | 1 | 0 | 0 | 1 | 1 | 1 | 0.016941 | 0.0256674 | 0.205339 | 8.267706 | 105.485 | 4.942792 |
| 1 | 37 | 61 | 2 | 24 | 1 | 2 | 4 | 0 | 0 | 0 | 0 | 0 | 1 | 0 | 0 | 1 | 1 | 0.029274 | 0.029274 | 0.263466 | 8.136226 | 104.796 | 0.399502 |
| 0 | 85 | 46 | 7 | 34 | 0 | 4 | 4 | 0 | 0 | 0 | 0 | 0 | 1 | 1 | 0 | 1 | 0 | 0.042538 | 0.0283585 | 0.129639 | 8.727616 | 110.112 | 2.063833 |
| 1 | 29 | 9 | 2 | 24 | 1 | 1 | 4 | 0 | 1 | 0 | 1 | 0 | 1 | 1 | 0 | 1 | 1 | 0.018335 | 0.020016 | 0.160128 | 8.516393 | 100.562 | 4.625199 |
| 0 | 37 | 63 | 2 | 27 | 0 | 4 | 4 | 0 | 0 | 0 | 0 | 0 | 1 | 0 | 0 | 1 | 0 | 0.018634 | 0.0186335 | 0.223603 | 8.300281 | 104.211 | 0.5670744 |
| 1 | 44 | 2 | 4 | 27 | 1 | 1 | 4 | 0 | 1 | 0 | 1 | 0 | 0 | 0 | 1 | 1 | 1 | 0.011522 | 0.0115221 | 0.138265 | 9.068662 | 107.4 | 11.53765 |
| 0 | 84 | 23 | 7 | 25 | 0 | 3 | 3 | 1 | 0 | 0 | 0 | 1 | 0 | 0 | 0 | 1 | 0 | 0.022454 | 0.017963 | 0.251482 | 8.624612 | 110.07 | 4.268024 |
| 1 | 64 | 66 | 8 | 32 | 1 | 2 | 1 | 1 | 0 | 0 | 0 | 0 | 1 | 0 | 0 | 1 | 1 | 0.00905 | 0.0217202 | 0.260643 | 8.434681 | 109.7 | -0.5144128 |
| 1 | 12 | 20 | 3 | 24 | 1 | 1 | 4 | 0 | 1 | 0 | 1 | 0 | 1 | 0 | 1 | 1 | 1 | 0.025468 | 0.025468 | 0.165542 | 8.968651 | 109.525 | -0.2710156 |
| 0 | 16 | 4 | 7 | 23 | 0 | 4 | 4 | 0 | 0 | 0 | 0 | 0 | 0 | 0 | 0 | 1 | 0 | 0.00898 | 0.0089799 | 0.197558 | 9.317939 | 106.413 | 3.110565 |
| 1 | 21 | 25 | 1 | 17 | 1 | 1 | 4 | 0 | 1 | 0 | 1 | 1 | 1 | 0 | 0 | 0 | 0 | 0.020194 | 0.0201939 | 0.201939 | 8.507546 | 104.375 | -1.034636 |
| 0 | 51 | 53 | 4 | 25 | 0 | 1 | 4 | 0 | 1 | 0 | 1 | 1 | 1 | 0 | 0 | 1 | 0 | 0.014037 | 0.0140371 | 0.196519 | 8.178078 | 107.939 | -0.5497003 |
| 0 | 70 | 44 | 6 | 28 | 1 | 4 | 1 | 0 | 0 | 0 | 0 | 0 | 1 | 1 | 0 | 1 | 0 | 0.030033 | 0.0150163 | 0.188776 | 8.670258 | 109.303 | 4.255041 |
| 1 | 47 | 64 | 4 | 34 | 1 | 3 | 4 | 0 | 0 | 0 | 0 | 0 | 0 | 0 | 0 | 0 | 0 | 0.019275 | 0.0192752 | 0.134927 | 8.554104 | 109.362 | 6.731958 |
| 1 | 29 | 5 | 2 | 40 | 1 | 1 | 4 | 0 | 1 | 0 | 1 | 1 | 1 | 0 | 0 | 1 | 1 | 0.018335 | 0.020016 | 0.160128 | 8.516393 | 100.562 | 4.625199 |
| 1 | 42 | 55 | 3 | 40 | 0 | 3 | 4 | 0 | 0 | 0 | 0 | 0 | 0 | 0 | 0 | 1 | 1 | 0.022298 | 0.0243427 | 0.121714 | 8.320691 | 106.225 | 0.3884559 |
| 1 | 15 | 25 | 5 | 38 | 1 | 4 | 4 | 0 | 0 | 0 | 0 | 1 | 1 | 0 | 0 | 1 | 1 | 0.01742 | 0.0174201 | 0.095811 | 9.348449 | 108.064 | 2.942514 |
| 0 | 13 | 39 | 5 | 18 | 1 | 1 | 4 | 0 | 1 | 0 | 1 | 0 | 0 | 0 | 0 | 1 | 0 | 0.012588 | 0.0125881 | 0.062941 | 8.980172 | 107.849 | 4.278709 |
| 0 | 14 | 36 | 5 | 31 | 1 | 3 | 4 | 0 | 0 | 1 | 1 | 0 | 1 | 0 | 0 | 1 | 0 | 0.017802 | 0.0089008 | 0.142412 | 9.326789 | 119.992 | 6.199605 |
| 0 | 52 | 20 | 4 | 21 | 1 | 1 | 4 | 0 | 1 | 0 | 1 | 0 | 1 | 1 | 0 | 0 | 0 | 0.02675 | 0.0133751 | 0.168144 | 8.785998 | 109.076 | 5.935247 |
